# Potato Production in Northwestern Europe (Germany, France, the Netherlands, United Kingdom, Belgium): Characteristics, Issues, Challenges and Opportunities

**DOI:** 10.1007/s11540-021-09535-8

**Published:** 2022-01-28

**Authors:** Jean-Pierre Goffart, Anton Haverkort, Michael Storey, Norbert Haase, Michel Martin, Pierre Lebrun, Daniel Ryckmans, Dominique Florins, Kürt Demeulemeester

**Affiliations:** 1 Walloon Agricultural Research Center (CRA-W), Gembloux, Belgium; 2 Department of Rural Sociology, Wageningen University and Research (WUR), Wageningen, the Netherlands; 3 Agriculture and Horticulture Development Board (AHDB), Kenilworth, UK; 4 OWL University of Applied Sciences and Arts, Lemgo, Germany; 5 ARVALIS - Institut du végétal, Estrées-Mons, France; 6 Filière Wallonne de la Pomme de Terre (FIWAP), Gembloux, Belgium; 7 Inagro, Rumbeke-Beitem, Belgium

**Keywords:** Industrial agri-food production, Production practices, Sustainability, Value-chain

## Abstract

In Northwestern Europe, Germany, France, the Netherlands, the UK and Belgium constitute the biggest five potato producers, with total potato crop production around 60% of EU-28 production before Brexit. Soil and climate conditions are highly favourable for potato growth in this region. Production is under driving forces of (i) the potato processing industry, particularly in Belgium; (ii) the innovation for fresh potato in the UK, France and Germany; (iii) the leadership of Germany and the Netherlands for starch potato; and (iv) the dominance of the Netherlands for seed production. Based on an industrial agri-food production system, the region has the highest potato yield levels worldwide and developed relevant trade networks for export of seed, fresh and processed potato products in and outside Europe. Conventional and intensive potato production is widespread over the region, whilst organic production started to develop in Germany and France. Whether the coming decades will be as successful as the last ones for sustainable potato production will depend on how the sector and stakeholders of the whole potato value-chain will overcome new issues and challenges. These are mainly soil quality and health conservation, consequences of climate change, increasing bans on the use of plant protection products, tightening environmental standards, food waste reduction and increasing trade tensions hampering the flow of potatoes around the world. After a detailed description of the potato production in the region, this paper contains a SWOT analysis aiming to identify potential solutions to overcome environmental, technical, economic, political and societal issues in the region for sustainable potato production in the coming years and decades.

## Introduction

Europe has a long tradition of potato production and use, initiated from its most likely introduction in Spain around 1570 through Spanish explorers, and its diffusion as an exotic gift a few years later to Italy, Belgium, Austria, and around 1600 to London, France and the Netherlands. From the seventeenth to the twentieth century, the potato crop developed progressively and to different degrees from a staple to a cash crop all over Europe (FAO 2009; Belgapom 2015). However, considering global potato production over the last 60 years, Europe shows a decrease by more than 50% whilst, over the same period, production shows a six-fold increase in Asia, a relatively lower increase in Africa and Latin America, whilst North America has remained at a similar level (FAO 2020). The same trends are observed regarding worldwide potato cropped areas. This decline in Europe has been caused, essentially, by a significant decrease in demand for fresh potatoes over recent decades. It is mainly due to its decreasing animal feed use in Eastern Europe where there has been progressive replacement by cereals, together with a shift of diets towards a wider range of foods and a trend towards convenience food requiring less preparation time in Western European countries (Devaux et al. 2020).

Despite this general decline in Europe, some Northwestern European countries (Belgium, France, Germany, the Netherlands, UK and Denmark) have increased or at least maintained over the last decade their potato cropped area and production (Eurostat 2019). The main driver in these countries is the huge growth, development and increasing international demand and trade (inside Europe and outside Europe to mainly Asia, Middle East and Latin America) for frozen processed potato products (Devaux et al. 2020). Although more limited, the increasing demand and trade for fresh potato to North African and Asian countries, together with seed and potato starch production, also contribute to the potato production level in these countries. In descending order, Germany, France, the Netherlands, UK and Belgium constituted the biggest five potato producers in the European Union, together with Poland and Romania (Eurostat 2020). Compared to the now ex EU-28 (including UK), these five countries annually mean-scored together over the last 10 years around 45% of the global European potato cropped area (0.81 versus 1.78 million hectares) and around 60% of the total European potato production (34.2 versus 57.0 million tonnes) (Eurostat 2019). Such values also illustrated higher average annual potato yield in these countries compared to EU-28 (42.2 versus 32.0 t ha^−1^), which are among the highest yield levels worldwide (Devaux et al. 2020). Such a relatively recent and successful development of potato production in these five Northwestern European countries raised new issues regarding its sustainable development in the future. Whether the coming decades will be as successful as the last ones will depend on how the potato production sector and stakeholders of the whole potato value-chain in Northwestern Europe will overcome new challenges. These include soil quality conservation; consequences of climate change; increasing bans (regulations and societal demands) on the use of chemical pesticides for weed, pest and disease control and tuber storage; tightening environmental standards; reducing food loss and waste; and increasing trade (import/export) tensions hampering the flow of potatoes around the world. Consequences of the COVID-19 pandemic overall sector will also need to be considered for the coming years.

This paper aims to describe, characterize and discuss the different topics, issues and challenges of the potato production in the five main Northwestern European countries (NWEC-05): Germany, France, the Netherlands, the UK and Belgium. It also focuses on solutions to maintain the sustainable development of potato production in this region. The first section describes a short history and the potato production areas characteristics (localization, soil and climate) in each of these countries. Main statistics and characteristics of either generic potato production or the different types of potato production in each country (consumption, starch and seed) will be presented and analyzed, including a description of the evolution of the production in relative amounts and values for conventional as well as organic production. Current economic and trade aspects of potato production in NWEC-05 are presented. Comparative situations at European and world scales are included for several topics where relevant. The second section will describe the different potato production practices currently developed in NWEC-05 from planting to storage, together with the technology level applied to the huge development of the processing industry and the necessary high-quality seed requirements. Based on a SWOT analysis of the potato production in NWEC-05, the third section will analyze the different issues and challenges facing the sector, whilst current and future drivers of the potato production will be identified and discussed, considering future prospects for a sustainable Northwestern European potato sector.

## Potato Production History, Characteristics, Statistics, Types, Economics and Environmental Footprint

### History

By the end of the fourth millennium B.C.E., most of the peripheral archipelagos of Northwestern Europe had been colonized by Neolithic farmers and exploited for animal husbandry and cereal growing (Encyclopedia.com 2020), a long time before potato introduction. According to FAO (2009), the relatively more recent introduction of potato in NWEC-05 is generally dated around the end of the sixteenth century and the beginning of the seventeenth century. In Germany, potato cultivation stayed initially low due to the cereal dominated three-field system (one or two parts cropped, the rest as fallow land). In Germany and France, it developed as a staple food crop not before the late eighteenth century, whilst in Belgium and the Netherlands the potato had become one of the country’s most important staple food crops within a century. In the UK, potato was being grown in London by 1597 and disseminated rapidly in Ireland but less so in England and Scotland. In a similar way in NWEC-05, potato production rose rapidly during the nineteenth and twentieth centuries and reached its maximum around 1960. The production is currently dedicated to the domestic fresh market, to the processing industry (French fries, crisps, frozen potato products, dehydrated products), starch production and to potato seeds, for domestic and export utilization, with different degrees of development within these five countries. Despite the common decline of potato production observed since 1960, they all rank among the world’s Top 20 potato producing countries (FAO, 2020), Germany, France and the Netherlands ranking among the Top 10.

### Characteristics of the Potato Production Areas in NWEC-05

#### Localization of Potato Cropped Areas in Regions Over NWEC-05

**Fig. 1:**
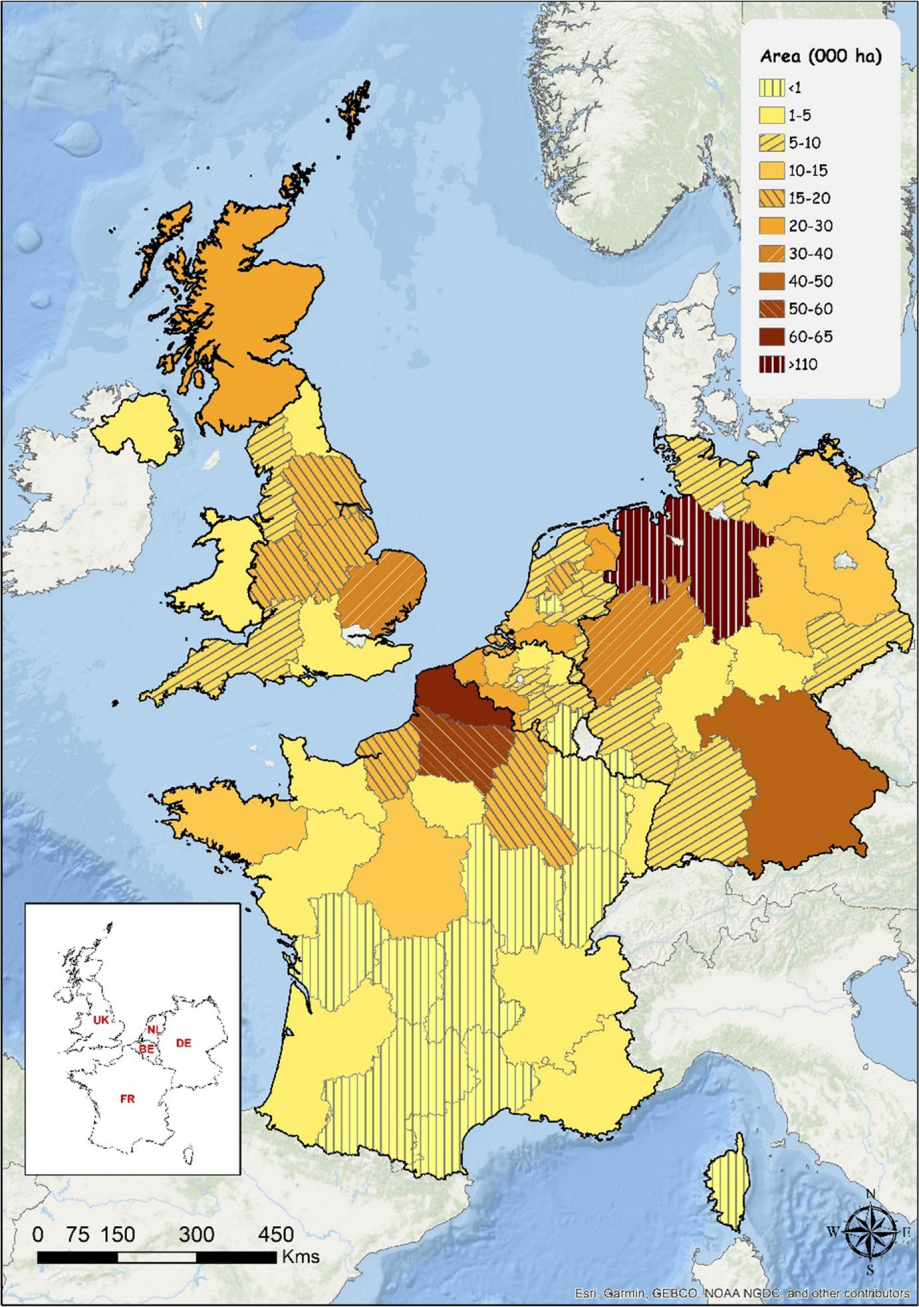
General potato cropped areas distribution (including seed) in the five main potato Northwestern European countries (NWEC-05) (average area value 2017, 2018, 2019) over 67 regions in Germany, France, the Netherlands, the UK and Belgium based on the 2016 European NUTS 2 classification (Nomenclature of Territorial Units for Statistics) (Source: Eurostat 2020, online data code: apro_cpshr, accessed 7 Jan 2021)

The distribution of potato cropped areas as average value for the years 2017, 2018 and 2019 over NWEC-05 is illustrated in Fig. 1. The regions represented on this map are based on the 2016 European Nomenclature of Territorial Units for Statistics (NUTS) classification at level 2 (https://ec.europa.eu/eurostat/web/nuts/background). This level corresponds to socio-economic analyses of basic regions for the application of regional policies. As illustrated from the map, most of the potato cropped area is located in the north half of the entire NWEC-05 zone. Specific hot spots are localized in northwest and southeast of Germany, north of France and to a degree, the midlands and east of the UK. In the Netherlands and Belgium, the potato cropped area distribution is more widespread all over the country.

In Germany, three main regions represent nearly 74% of the German potato cropped area: Lower Saxony (115,300 ha) and North-Rhine Westphalia (34,900 ha) in the northwest of the country and Bavaria (40,500 ha) in the south. Three other regions in the northeast represent 15% of the production: Saxony-Anhalt (14,500 ha), Mecklenburg-Western Pomerania (12,500 ha) and Brandenburg (10,600 ha). Four other regions represent nearly 10% of the potato cropped area: Rhineland Palatinate, Saxony, Schleswig-Holstein and Baden Wurttemberg, each with an area ranging from 5000 to 7000 ha. Thuringia and Hessen regions have an area ranging from 1000 to 4000 ha, and Saarland less than 1000 ha.

In France, most of the potato production area is located in the north half of the country. Two leading regions of the Hauts de France in the north represent nearly 60% of the potato cropped area: Nord-Pas-de-Calais (62,800 ha) and Picardy (56,300 ha). Champagne (18,600 ha) in the northeast and Upper Normandy (16,900 ha) in the northwest together represent another 18%, whilst Center-Loire Valley (13,600 ha) and Brittany (12,000 ha) in the extreme west represent nearly 13% of the French potato cropped area. In descending order, seven other regions represent nearly 8% of the area with values ranging from 1000 to 5000 ha: Ile-de-France (5000 ha), Aquitaine (3400 ha), Rhône-Alpes (2000 ha), Lower Normandy (1600 ha), Pays-de-la-Loire (1300 ha), Alsace (1200 ha) and Provence-Alpes-Côte d’Azur (1000 ha). The remaining regions are poor contributors with areas lower than 1000 ha.

In the Netherlands, potato production is widespread over the different regions of the country. Five leading regions represent 70% of the potato cropped area: Drenthe (28,600 ha) and Groningen (27,000 ha) in north Netherland mainly for starch potato production and close to Lower Saxony German region, North Brabant (21,200 ha) in south Netherlands, Zeeland (19,000 ha) in west Netherlands and Flevoland (18,700 ha) in the centre of Netherlands. Six other regions represent the remaining 30% with values ranging from 5000 to 10,000 ha: South Holland (10,000 ha) and North Holland (9500 ha) in west Netherlands, Friesland (9000 ha) in north Netherlands, Overijsel (7400 ha) and Gelderland (5900 ha) in east Netherlands and Limburg (7100 ha) in south Netherlands. Region Utrecht is a poor contributor.

In the UK, potato production is also more widespread over several regions. However, two main regions represent 46% of the UK potato cropped area: East of England (37,100 ha) and Scotland (28,300 ha) which produces the majority of UK seed potatoes. Another 35% of the potato cropped area is composed of three regions: Yorkshire and the Humber (18,800 ha), East Midlands (16,700 ha) and West Midlands (15,000 ha). North West (7400 ha) and South West (8000 ha) regions together represent around 11%. The four remaining lower contributing regions with areas ranging from 1000 to 5000 ha are in descending order: Northern Ireland (3900 ha), South East (3240 ha), Wales (3170 ha) and North East (1300 ha).

In Belgium, a large part of the potato production is located in the western part of the country close to the Nord-Pas-de Calais area in France. Two main regions represent 50% of the Belgian potato cropped area: West Flanders (25,700 ha) in the Flemish region and Hainaut (21,700 ha) in the Walloon region. Other secondary contributing regions with a combined 41% are East Flanders (12,700 ha) and Flemish Brabant (6300 ha) in the north part of the country, and Walloon Brabant (7100 ha), Liège (6850 ha) and Namur (6230 ha) in the south part. Regions Antwerp (4370 ha) and Limburg (3300 ha) in the north are lower contributors. Region Luxemburg in the south has the lowest contribution.

#### Potato Shares of Arable Land Area

**Fig. 2:**
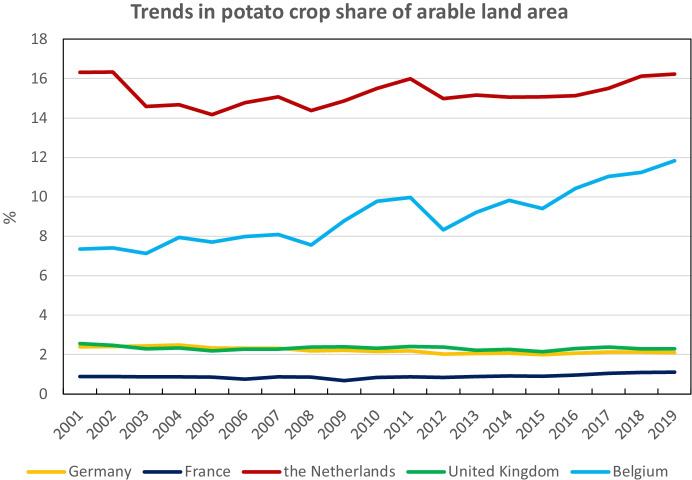
General potato crop share (including seed) of arable land area from 2001 to 2019 in the five main potato Northwestern European countries (NWEC-05) (Source: Eurostat 2020, online data code: apro_cpsh1, accessed 29 Oct 2020)

For the two last decades (2001 to 2019) as illustrated in Fig. 2, the current levels and trends of total potato crop share of arable land area by country over the NWEC-05 show large differences. With respective share values of around 16% and 12% in 2019, the Netherlands and Belgium have a relatively higher potato share of arable land area than UK (2.3%), Germany (2.1%) and France (1.1%), mainly due to the relatively higher territorial size of these last countries and the importance of their potato production. Whilst these three last countries have kept these proportions over the last two decades, the Netherlands share has fluctuated between 14 and 16% during the same period, whilst Belgium has considerably increased its share from 7.3 to 11.8%.

#### Agricultural Holdings Producing Potatoes

Based on European 2016 official data (Eurostat 2020), Germany and France have the highest number of holdings producing potatoes within the NWEC-05, respectively around 29,000 and 24,800, with an average potato cropped area/holding around 7 to 8 ha (Table 1). It is clearly contrasting with the three times lower number of holdings in the Netherlands and UK with twice the average cropped area (around 16 ha), that is mainly explained through the much lower number of holdings with a potato cropped area lower than 5 ha in these countries. Belgium officially scores a higher number of holdings than the Netherlands and UK, but with the lowest average potato cropped area/holding in NWEC-05, also due to a higher holdings number with harvested area lower than 5 ha. Such values illustrate quite different distributions of potato cropped area/holding between NWEC-05. In comparison to EU-28 data, there is a huge difference with the NWEC-05, either as average potato area/holding or as distribution of cropped area/holding.

**Table 1:** Characteristics of agricultural holdings producing potatoes in NWEC-05 compared to EU-28 (year 2016)

Country/Region	Holdings producing potatoes	Cropped potato areas	Average potato area/holding	Holdings with potato area < 5 ha	Holdings with potato area > 5 ha
	Number	ha	ha	Number	Number
Germany	28,910	242,500	8.4	19,660	9250
France	24,780	179,000	7.2	14,870	9910
The Netherlands	9570	155,590	16.3	2100	7470
United Kingdom	8390	139,000	16.6	2140	6250
Belgium	12,880	89,210	6.9	5330	7550
**NWEC-05**	**84,530**	**805,300**	**9.5**	**44,100**	**40,430**
EU-28	1,497,880	1,689,400	1.1	1,433,640	64,240

Sources: Eurostat 2020 (Eurostat, online data code: ef_lac_rootcrop, year 2016)

The European data set on holdings producing potatoes is based on Common Agricultural Policy (CAP) declarations. However, this CAP holding number does not properly reflect the much lower numbers of commercially significant growers producing the majority of the NWEC-05 crop. In Belgium, the number of true potato holdings is estimated around only 6000 to 8000 (Pierre Lebrun, personal communication, 2021), quite lower than the official number, mainly because of high land rental costs being driven by the dominant processing industry. In France, a similar number of holdings produce more than 80% of the total production. Similarly, in the UK, many small potato holdings are officially registered but their contribution to national tonnage is very limited and usually for on-farm/home use and less than 1700 grower businesses produce the majority of the crop. In Germany, the number of holdings with low potato production is declining because of high mechanization costs and it has become a question of either intensified production or stopping potato growing.

#### Climate and Soil Characteristics Within Each Potato Production Area

##### Past and current situation

Most of the areas for potato crop in NWEC-05 are composed of naturally loose and deep soils, which are ideal to allow deep soil preparation and easy enlargement of the tubers. Potato soil types in NWEC-05 are generally loamy and sandy loam that are well supplied in organic matter, with good drainage and aeration, with a pH ranging from 5.0 to 7.0 (Table 2). Such soil conditions are the most suitable for the potato crop growth.

**Table 2:** Main soil types and average climatic conditions for potato cropping areas in NWEC-05

Country	Main soil type	Soil organic carbon content (SOC)	pH	Average annual temperature		Average annual rainfalls
		%		min (°C)	max (°C)	mm
Germany^1^	Sandy	1.0	5.5	6	13	571
	Sandy loam	1.6	6.5			
France^2^	Loamy clay	1.6 to 1.8	6.5 to 8.5	9	16	637
The Netherlands^3^	NE Sand/Peat	10–20	5.0	6	14	838
	NW Marine clay	2.0	7.0			
United Kingdom^4^	Sandy/sandy loam	1.7	7.1	8	15	557
	Clay loam/clay	3.8	7.5			
Belgium^5^	Sandy/sandy loam	1.1 to 1.6	6.0 to 8.0	7	14	852
	Loamy/clay loam					

Soil data sources:

^1^https://www.umweltbundesamt.de/publikationen/erarbeitung-fachlicher-rechtlicher-0, accessed Feb. 2021; pH: https://www.bmel.de/DE/themen/landwirtschaft/pflanzenbau/bodenschutz/bodenfruchtbarkeit-kalkung-grundlagen.html, accessed Feb. 2021

^2^https://www.gissol.fr/donnees/cartes

^3^https://www.knmi.nl/klimaat-viewer/kaarten/neerslag-verdamping/gemiddelde-hoeveelheid-neerslag/jaar/Periode%201991-2020

^4^https://www.niab.com/research/agronomy-and-farming-systems/potatoes/grower-platform-resilient-rotations and https://www.cawoodscientific.uk.com/nrm

^5^https://www.requasud.be/publications/; https://www.bdb.be/Info/Publicaties/tabid/100/language/nl-BE/Default.aspx

Climate data sources:

https://www.currentresults.com/Weather/Europe/Cities/temperature-annual-average.php

https://www.currentresults.com/Weather/Europe/Cities/precipitation-annual-average.php

^1^Berlin, ^2^Paris, ^3^Amsterdam, ^4^London, ^5^Brussels

The climate conditions in NWEC-05 are optimal as the potato is a cool weather crop, temperature being the main limiting factor to production: optimum yields are obtained where mean daily temperatures range between 18 and 20 °C, crop growth being sharply inhibited in temperatures below 10 °C and above 30 °C (Haverkort et al. 2015). Regular water supply is also required to maintain soil moisture at a relatively high level for optimal growth of the crop. As a 120 to 150 days crop (as mostly encountered in NWEC-05), the potato requires from 500 to 700 mm of water, and a significant part of the crop water demand is covered with normal local precipitation level from April to October in NWEC-05 (Table 2). However, to reach a high yield level, supplementary irrigation is generally required to improve yield response by around 10 to 20% in most of the five countries. However, for the two last decades, climate change with increasing temperature and increasing occurrence of summer extreme weather events such as heat waves and drought (as in 2003, 2006, 2007, 2010, 2014, 2015; from AgriAdapt (2017) and also more recently (2018, 2019)), or regional heavy precipitation and flooding (2018) and sometimes severe hail storms, potato growth can be locally or more widely adversely affected. Irrigation in most potato areas in the NWEC-05 is becoming more crucial to maintain stable yields (Janssens et al. 2020). Furthermore, storing potatoes is increasingly complicated and expensive as due to heat peaks in summer and warmer weather between autumn and spring, tubers tend to break dormancy and sprout earlier and quicker than they used to do a decade ago.

##### Prospect on Future Climate Change and Evolution for the Coming Decades with Potential Effects on the Potato Crop in NWEC-05

There is a strong trend towards wetter autumns and winters but drier springs and summers. Summer and winter temperatures have risen by about 1 °C since 1950 and are likely to increase by another degree C in the next 30 years (IPCC 2021). This means that the frost-free period is prolonged by 2 weeks in spring and 2 weeks in autumn. With an increase of the carbon dioxide (CO_2_) concentration from 360 to 600 ppm over the same period, its photosynthesis enhancing effect increases the growth rate by about 25% (Jaggard et al. 2010). According to Haverkort et al. (2015), when applying a potato crop growth model and inserting new planting and harvest dates, and the increased solar radiation use efficiency, this would lead to a yield increase over 30% over the coming 30 years. So, where growers now attain 60 t ha^−1^, the new yields theoretically will be about 80 t ha^−1^ in 2050. However, wetter winters do not make the soil accessible for cultivation any earlier and the increased frequency of heat waves in summer are likely to reduce the gain in growth rate and will affect tuber quality with a trend to lower dry matter concentration depending on cultivar sensitivity to secondary growth. Adaptation measures to benefit from the climate change therefore need to involve mechanization to cope with wetter planting and harvesting conditions (lighter machinery) and irrigation practices that cope with the increased frequency of dry spells to supply water and to cool the crop during heat waves. With more and more areas becoming too hot and dry for production of potatoes in South and some Eastern Europe regions, the relative importance of production in Northwestern Europe is likely to increase. There will also be changes in the distribution and development of pests and pathogens that affect the crop (Haverkort and Verhagen 2008; Haverkort et al. 2013a).

### Potato Production Statistics in NWEC-05

Among the NWEC-05, Germany is currently the biggest potato producer (including seed) with average annual potato production for the period 2017–2019 of just over 10.4 million tonnes (Table 3). Thereafter in descending order are France (8.3 M tonnes), the Netherlands (6.8 M tonnes), UK (5.5 M tonnes) and Belgium (3.8 M tonnes). The five countries range similarly regarding the average annual potato cropped areas, from 258,100 ha for Germany to 94,800 ha for Belgium (Table 3). Estimated average annual yields between countries for the same period indicate quite small differences, ranging from 38.4 t ha^−1^ in the UK to 41.5 t ha^−1^ in the Netherlands. The annual total potato production in NWEC-05 with an average value of 34,870 million tonnes for the period 2017–2019 (Table 3) represents 61.5% of the EU-28 production, whilst the average annual cropped areas for the same period (859,900 ha) represents 49.6% of the EU-28 harvested area (Table 3). The part of EU-28 production by country is 18.4% for Germany, 14.7% for France, 12.0% for the Netherlands, 9.7% for the UK and 6.8% for Belgium. The distribution share of EU-28 cropped areas is similar with values of respectively 14.9%, 11.6%, 9.4%, 8.2% and 5.5%.

**Table 3:** Potato production indicators (production, cropped area and yield; including seed) in the five main potato producers of Northwestern European countries (NWEC-05), compared to EU-28, for the period 2017-2019 and average annual growth rate for the period 2001–2019

Country/Region	2017–2019 (3y-avge)			Average annual growth rate					
				2017–2019 vs. 2001-2008 (8y-avge)			2017–2019 vs. 2009–2016 (8y-avge)		
	Production	Area	Yield	Production	Area	Yield	Production	Area	Yield
	(× 000 t)	(× 000 ha)	(t ha^−1^)	%	%	%	%	%	%
Germany	10,414.3	258.1	40.4	− 7.7	− 7.6	0	− 4.0	**4.2**	− 7.8
France	8322.7	200.3	41.6	**24.4**	**26.1**	− 1.2	**17.5**	**22.4**	− 3.9
The Netherlands	6792.8	163.7	41.5	− 1.6	**3.0**	− 4.4	− 1.2	**5.2**	− 6.1
United Kingdom	5510.0	143.0	38.4	− 10.5	− 2.9	− 8.1	− 3.3	**1.7**	− 5.4
Belgium	3829.9	94.8	40.4	**34.8**	**47.6**	− 8.6	**8.2**	**20.6**	− 10.4
Total NWEC-05	34,869.8	859.9	40.6	**2.9**	**6.3**	− 3.1	**2.4**	**9.4**	− 6.2
EU-28	56,743.8	1733.5	32.7	− 13.4	− 28.9	**21.1**	− 0.8	− 3.8	**2.8**
EU-28 (excl.NWEC-05)	21,874.0	873.6	25.0	− 30.8	− 46.4	**28.2**	− 5.5	− 14.1	**9.2**

Source: Eurostat 2020, Eurostat (online data code: apro_cpsh1), accessed 29 Oct 2020

As indicated in Table 4 for the period 2017–2018, the potato production share of NWEC-05 within Europe is 31.4%, and 9.3% at world scale. The total potato cropped area share of NWEC-05 within Europe is 17.7% and 4.8% at world scale. Yields in NWEC-05 are around twice the European and World levels.

**Table 4:** Potato production indicators (production, cropped area, yield; including seed) in NWEC-05 compared to Europe and world as average values for the period 2017–2018

Region	Production	Cropped area	Yield	Europe production share	Europe cropped area share	World production share	World cropped area share
	(× 000 t)	(× 000 ha)	t ha^−1^	%	%	%	%
NWEC-05^1^	34,587.0	846.5	40.9	31.4	17.7	9.3	4.8
Europe^2^	110,115.0	4793.4	23.0	100	100	29.7	27.2
World^2^	370,971.6	17,601.2	21.1	–	–	100	100

^1^Eurostat 2020, Eurostat (online data code: apro_cpsh1), accessed 29 Oct 2020

^2^FAO, 2020http://www.fao.org/faostat/en/#search/Potatoes, accessed 17 Dec 2020

**Fig. 3:**
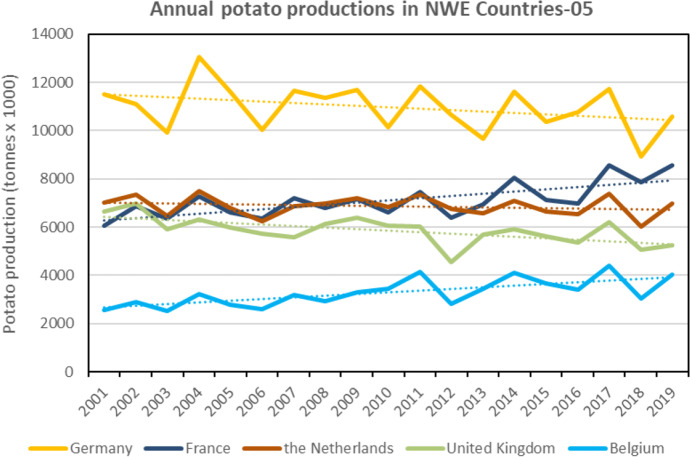
Total annual potato production (including seed) from 2001 to 2019 in the five main potato Northwestern European countries (NWEC-05). Dotted lines represent trend in evolution for each country. (Source: Eurostat 2020, Eurostat (online data code: apro_cpsh1), accessed 29 Oct 2020)

**Fig. 4:**
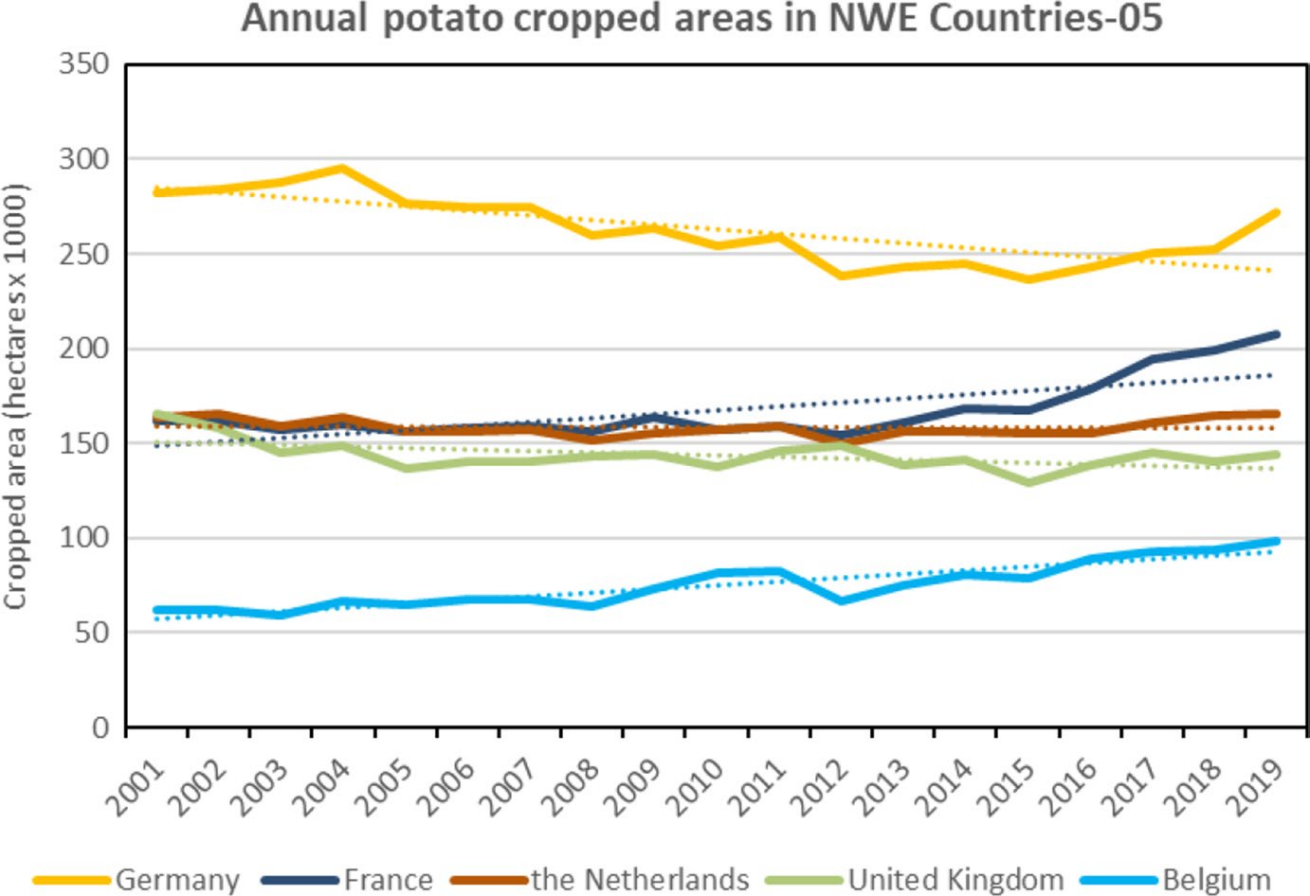
Total annual potato cropped area (including seed) from 2001 to 2019 in the five main potato Northwestern European countries (NWEC-05). Dotted lines represent trend in evolution for each country (Source: Eurostat 2020, Eurostat (online data code: apro_cpsh1), accessed 29 Oct 2020)

Whilst potato production has been drastically falling since 1960, the evolution of total annual potato productions and cropped areas over the last two decades (from 2001 to 2019) indicate different trends over NWEC-05. Average annual growth rate is still decreasing for potato production in Germany, the Netherlands and UK, whilst it is increasing in France and Belgium. Comparing the average value for the periods 2017–2019 vs. 2001–2008, Table 3 indicates growth rate values of 24.4% and 34.8% for France and Belgium respectively, whilst negative rates are observed for the UK (− 10.5%), Germany (− 7.7%) and the Netherlands (− 1.6%). Similar trend is observed when comparing periods 2017–2019 vs. 2009–2016, but at a lower extent. For the total average annual cropped areas (Table 3), similar trends are observed when comparing period 2017–2019 vs. 2001–2008, whilst for the periods 2017–2019 vs. 2009–2016, cropped areas are increasing in all five countries, but mainly in France and Belgium with average growth rates of 22.4% and 20.6% respectively, and with 4.2% for Germany, 5.2% for the Netherlands and 1.7% for the UK. Clearly, France and Belgium have dramatically increased their potato production and cropped areas over the last decade, starting 2009 in Belgium and 2013 in France (Figs. 3 and 4), whilst Germany, the Netherlands and the UK have increased their cropped area only for a few recent years over the last decade, but not their production (Table 3, Figs 3 and 4). The development of the potato area first in Belgium, then in France is mainly due to ever higher needs of potato processors in Belgium and to a lesser extent to the development of French exports of table potatoes (initially to Spain). Considering the NWEC-05 overall, total potato productions and cropped areas increased, comparatively to both periods respectively, with average growth rate of 2.9% and 2.4% for potato productions, and 6.3% and 9.4% for cropped areas (Table 3).

**Fig. 5:**
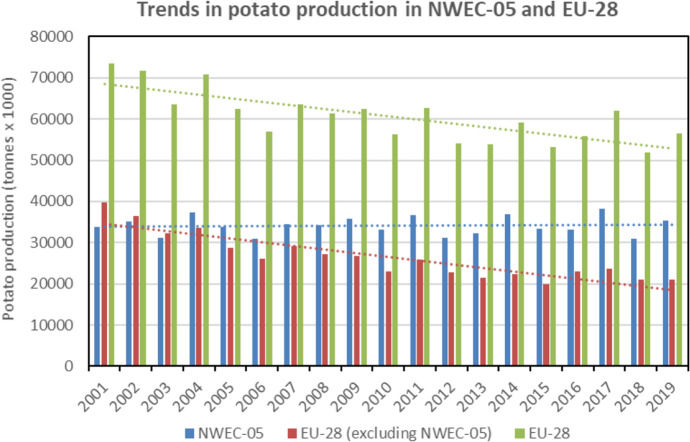
Comparison of total potato production (including seed) in NWEC-05 and EU-28 from 2001 to 2019. Dotted lines represent trend in evolution for each group of country (Source: Eurostat 2020, Eurostat (online data code: apro_cpsh1) accessed 29 Oct 2020)

**Fig. 6:**
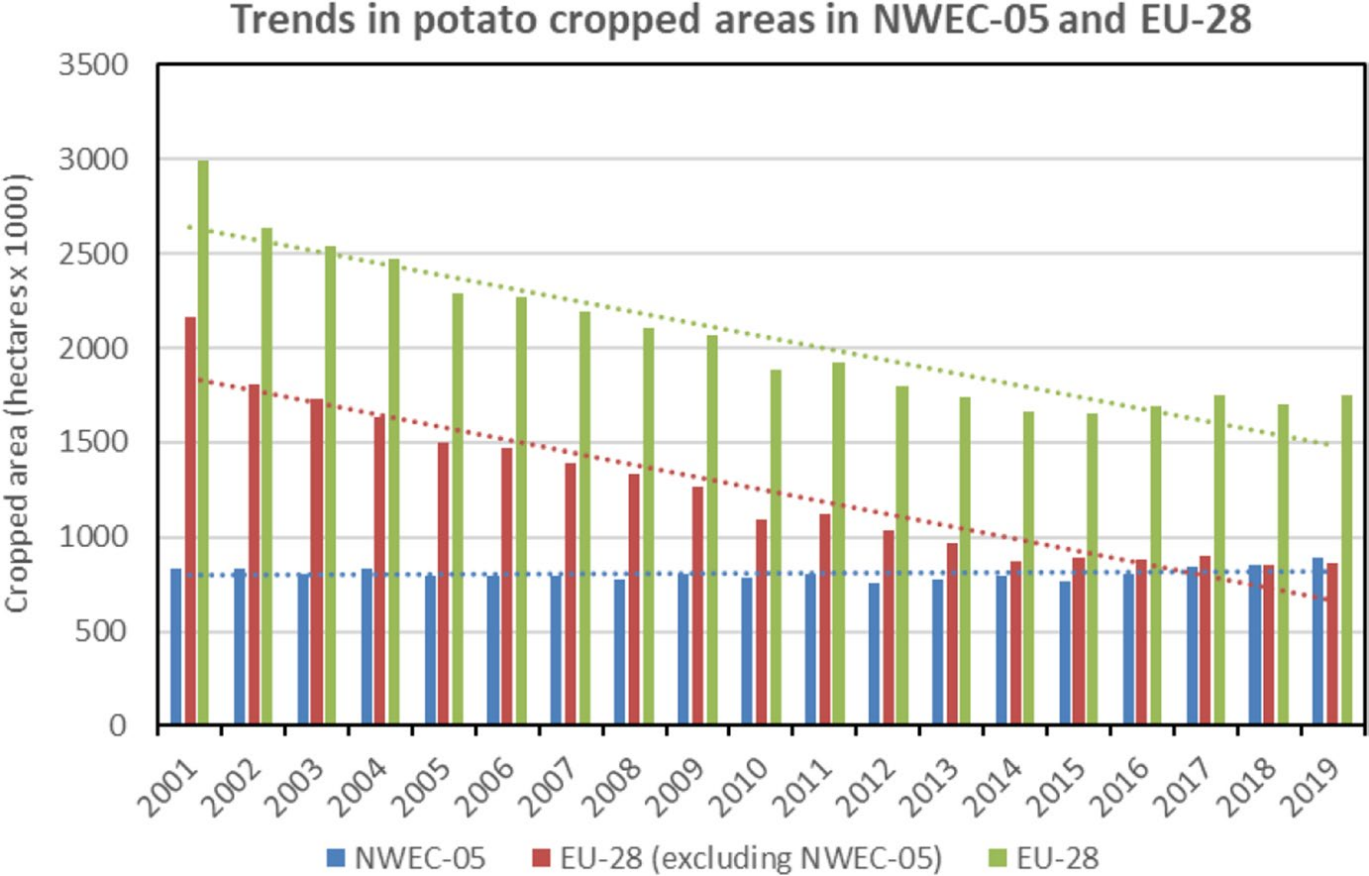
Comparison of total potato cropped areas (including seed) in NWEC-05 and EU-28 from 2001 to 2019. Dotted lines represent trend in evolution for each group of country (Source: Eurostat 2020, Eurostat (online data code: apro_cpsh1) accessed 29 Oct 2020)

**Fig. 7:**
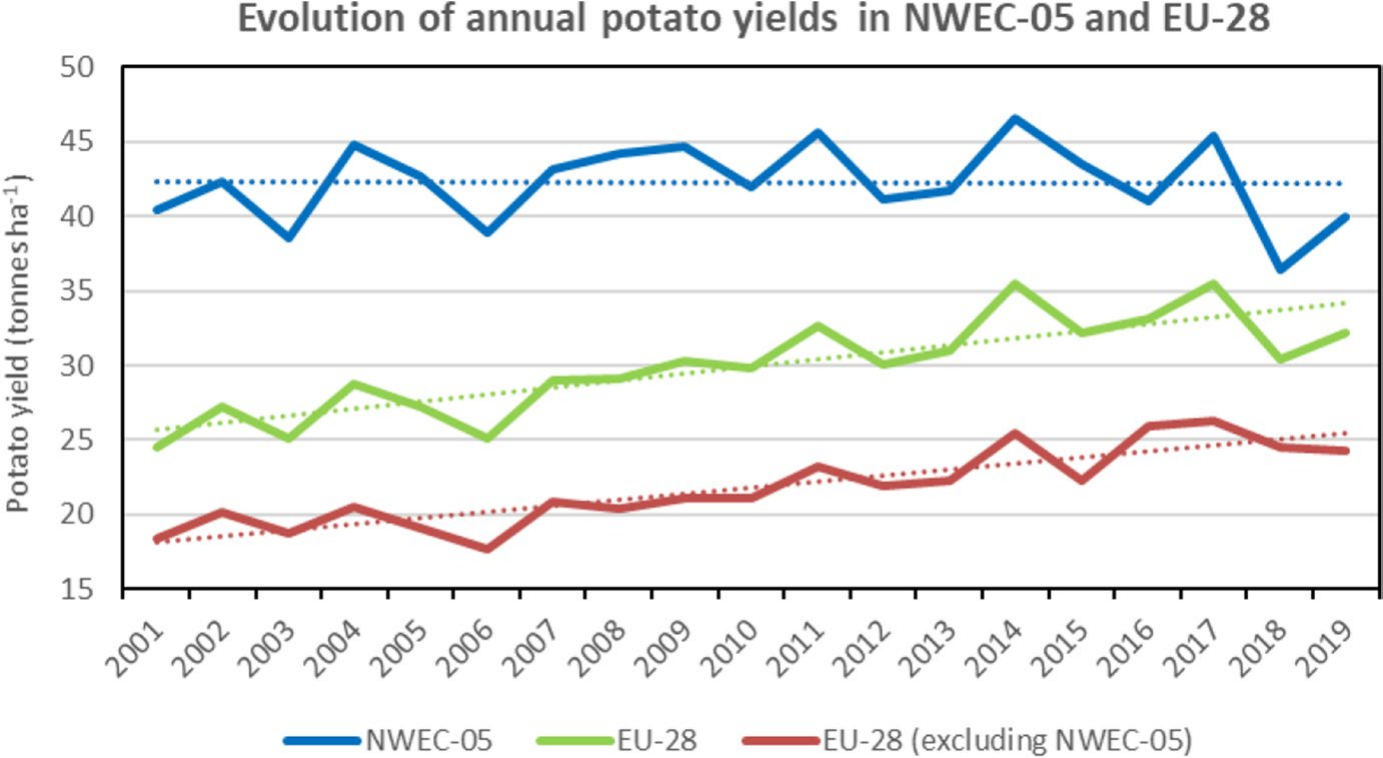
Evolution and trends in annual potato yields in NWEC-05 and EU-28. Dotted lines represent trend in evolution for each group of country (Source: estimated from Eurostat 2020, accessed 29 Oct 2020)

In comparison to EU-28 countries (excluding the NWEC-05), the trends in NWEC-05 contrast dramatically with decreasing potato production (Fig. 5) and cropped areas (Fig. 6) within the rest of EU-28. Comparative to the period 2017–2019, it is mainly over the period 2001–2008 that significant decreases were observed in the remaining EU-28 with values of − 30.8% for annual potato production (Table 3) and − 46.4% for annual cropped areas, whilst values were respectively of − 5.5% and − 14.1% for the period 2009–2016, indicating a much slower decrease for this period. It is also important to note that over the two last decades, yields in EU-28 and in EU-28 excluding NWEC-05 were clearly increasing, whilst the trend in NWEC-05 remained stable (Fig. 7). Therefore, total yield increase in EU-28 is explained by the yield average annual growth rates for the part of EU-28 excluding NWEC-05 with values of 28.2% and 9.2% when comparing 2017–2019 period respectively to 2001–2008 and 2009–2016 periods (Table 3). Whilst the trend for yield over the two last decades period in NWEC-05 appeared stable in Fig. 7, growth rate values for yield in Table 3 for each of the NWEC-05 are decreasing, more particularly for Belgium. Altogether, the NWEC-05 averaged a decrease of 3.1% comparatively to the period 2001–2008, and of 6.2% comparatively to the period 2009–2016 (Table 3). This is mainly explained by the particularly very bad summer weather conditions in Northwestern Europe in 2018 that significantly hampered yield, as illustrated in Fig. 7, and induced a low average yield for the reference period 2017–2019. Increasing cropped areas in NWEC-05 largely contributed to compensate for yield decrease in terms of total production in 2018 over the region. However, this apparent trend of declining yields observed in recent years in NWEC-05 should be monitored in the future. Surprisingly for the rest of EU-28, yields are clearly increasing over the two last decades (Fig. 7). Average annual growth rates of yield for the rest of EU-28 are 28.2% and 9.2% when comparing 2017–2019 period to 2001–2008 and 2009–2016 periods respectively (Table 3).

### Types of Potato Productions in NWEC-05

In NWEC-05, potato production covers potato consumption for fresh market and food processing industry, including early potato production, starch potato and seed. Limited other uses are not mentioned here (e.g. animal feed or ethanol production). These types are mainly produced under conventional and intensive cropping practices. However, an increasing proportion is produced under organic agricultural practices.

#### Consumption, Starch and Seed Productions Under Conventional Agriculture

As illustrated in Tables 5 and 6, the current potato cropped areas for production of consumption, starch and certified seed potato vary largely within NWEC-05.

**Table 5:** Potato cropped areas for consumption, starch and certified seed production by country within NWEC-05 under conventional agriculture (average annual data for the period 2017–2019)

Country/Region	Consumption^1^			Starch			Certified seed		
	Cropped area	Share in the country	*Share of NWEC-05*	Cropped area	Share in the country	*Share of NWEC-05*	Cropped area	Share in the country	*Share of NWEC-05*
	x 000 ha	%	***%***	x 000 ha	%	***%***	x 000 ha	%	***%***
Germany^a^	185.2	71.8	***30.1***	53.2	20.6	***43.9***	16.3	6.6	***16.3***
France^b^	154.6	77.3	***25.1***	23.3	11.6	***19.2***	22.2	11.1	***22.2***
The Netherlands^c^	76.1	46.5	***12.4***	44.7	27.3	***36.9***	42.9	26.2	***42.7***
United Kingdom^d^	107.7	87.0	***17.5***	0	0	***0***	16.1	13.0	***16.1***
Belgium^e^	92.3	97.6	***15.0***	0	0	***0***	2.5	2.4	***2.5***
**NWEC-05**	615.9		*100*	121.2		*100*	100.0		*100*

^1^Consumption potato for fresh market and food processing industry, including early potato production

^a^https://www.ble.de/DE/BZL/Daten-Berichte/Kartoffeln/Kartoffeln_node.html, accessed Feb. 2021; https://www.bundessortenamt.de/bsa/sorten/beschreibende-sortenlisten/download-bsl-im-pdf-format; accessed Feb. 2021; note: compared to global potato cropped area value in Table 3, 2700 ha are not mentioned here as it is used for other purposes, e.g. fodder

^b^https://agreste.agriculture.gouv.fr/agreste-saiku ; https://www.gnis.fr/etudes-donnees-statistiques-semences/, accessed Dec 2020

^c^https://www.cbs.nl/en-gb/figures/detail/7100eng, accessed Dec 2020

^d^https://ahdb.org.uk/potato/planted-area-sector, accessed Dec 2020—https://www.gov.uk/government/statistics/statistical-review-of-northern-ireland-agriculture-2019 (accessed Jan 2021); note: compared to global potato cropped area value in Table 3, the mentioned value relates to harvested area, and therefore some circa 20,000 ha are not mentioned

^e^Statbel - https://statbel.fgov.be

**Table 6:** Potato production for consumption, starch and certified seed by country within NWEC-05 under conventional agriculture (average data for the period 2017–2019)

Country/Region	Consumption^1^			Starch			Certified Seed		
	Production	Share in the country	*Share of NWEC-05*	Production	Share in the country	*Share of NWEC-05*	Production	Share in the country	*Share of NWEC-05*
	x 000 tonnes	%	***%***	x 000 tonnes	%	***%***	x 000 tonnes	%	***%***
Germany^a^	6523.1	62.6	***25.7***	2080.0	20.0	***43.3***	548.0	5.2	***15.9***
France^b^	6543.5	78.6	***25.8***	1011.2	12.1	***21.0***	768.0	9.2	***22.2***
The Netherlands^c^	3599.7	53.0	***14.2***	1713.0	25.2	***35.7***	1480.1	21.8	***42.9***
United Kingdom^d^	4920.2	89.3	***19.4***	0	0	***0***	589.8	10.7	***17.1***
Belgium^e^	3762.7	98.2	***14.8***	0	0	***0***	67.2	1.8	***1.9***
**NWEC-05**	25,349.2	100	*100*	4804.2		*100*	3412.4	100	*100*

^1^Consumption potato for fresh market and food processing industry, including early potato production

^a^https://www.bmel-statistik.de/archiv/statistisches-jahrbuch/, accessed Feb. 2021; https://www.ble.de/DE/BZL/Daten-Berichte/Kartoffeln/Kartoffeln_node.html, accessed Feb 2021; note: compared to global production mentioned in Table 3, values mentioned here concern data without shrinkage and other uses (e.g. fodder)

^b^https://agreste.agriculture.gouv.fr/agreste-saiku; https://www.gnis.fr/etudes-donnees-statistiques-semences/, accessed Dec 2020

^c^https://www.cbs.nl/en-gb/figures/detail/7100eng, accessed Dec 2020

^d^https://ahdb.org.uk/potato/production, accessed Dec 2020 ; https://www.gov.uk/government/statistics/statistical-review-of-northern-ireland-agriculture-2019 (accessed Jan 2021)

^e^Statbel: https://statbel.fgov.be

In Germany, a major part (71.8%) of the cropped area (Table 5) and 62.6% of the production (Table 6) is for consumption potatoes, whilst around 21% is for starch production and 5% for seed production, based upon area and production (overall storage losses for food production and other uses (e.g. feed) are not considered here). Germany is leader within NWEC-05 for starch potato production, with a share for production of 43.3%, whilst the share production for consumption potato is quite similar to France and the highest within NWEC-05 with 25.7%. Among the 10 most important potato varieties (Table 7), there are four varieties for starch production (Kuras, Eurogrande, Axion, Novano), two varieties for fresh consumption (Belana, Annabelle) and four varieties for processing (Fontane, Gala, Agria, Verdi). The market for fresh consumption is not dominated by only a few varieties, but is more diverse with 164 varieties, covering different maturity classes (from very early to late) and different boiling behaviour (firm to soft). In total, the German list of varieties includes 228 varieties (BSA 2020). During the last six decades, consumers have changed their preferences from table potatoes towards prefabricated potato products. In 2017, the per capita consumption was 60.4 kg; however, fresh consumption was only 23.6 kg (BLE 2019). Production of starch potatoes is closely linked with the demand of the potato starch industry. The competition with other starch sources and the future perspective of optimized equipment use (field production) will determine future shares of that type of potato production.

**Table 7:** Top-10 varieties and relative cropped areas in NWEC-05 for consumption potato (fresh market and food processing, including early potato varieties) and starch production under conventional agriculture (average annual data for period 2017–2019)

Germany^a^		France^b^		The Netherlands^c^		United Kingdom^d^		Belgium^e^	
Variety (all production types)	Area %	Variety (seed production only^1^)	Area %	Variety (seed production only^1^)	Area %	Variety (all production types)	Area %	Variety (all production types)	Area %
Belana^2^	6.8	Spunta^2^	8.6	Spunta^2^	11.7	Maris Piper^2,^,^1F^	11.8	Fontane^1F^	35.1
Fontane^1F^	6.7	Fontane^1F^	5.6	Fontane^1F^	10.4	Markies^1F, 2^	5.0	Bintje^1F^	17.3
Gala^1F, 2^	5.2	Innovator^1F^	4.0	Agria^1F^	5.8	Maris Peer^2^	3.9	Innovator^1F^	10.1
Kuras^1S^	3.8	Agata^2^	3.6	Innovator^1F^	5.6	Melody^2^	3.3	Challenger^1F^	8.9
Agria^1F, 2^	3.3	Challenger^1F^	2.9	Arizona^2^	2.9	Lady Rosetta^1C^	3.0	Markies^1F^	4.3
Verdi^1C^	2.4	Amyla^1S^	2.7	Fabula^2^	2.1	Nectar^2^	2.8	Royal^1F^	2.4
Annabelle^2^	2.0	Kaptah Vandel^1S^	2.6	Markies^1F, 2^	1.8	Taurus^1C^	2.7	VR808^1C^	1.6
Eurogrande^1S^	2.0	Markies^1F, 2^	2.4	Challenger^1F^	1.8	Innovator^1F^	2.4	Felsina^1F^	1.4
Axion^1S^	1.9	Monalisa^2^	2.2	Columba^2^	1.6	Royal^1F^	2.3	Lady Claire^1C^	1.2
Novano^1S^	1.5	Bintje^1F^	1.2	Manitou^2^	1.3	Sagitta^1F, 2^	2.2	Lady Anna^1F^	1.1
Other varieties.	64.4	Other varieties.	64.2	Other varieties.	55.0	Other varieties.	60.1	Other varieties.	16.6
100%~258,100 ha		100%~22,200 ha		100%~42,900 ha		100%~143,000 ha		100%~96,000 ha	

Main use: ^1^Food processing: ^F^French Fries, ^C^Crisps, ^S^Starch; ^2^Fresh market

Source of data:

^a^https://www.bmel-statistik.de/landwirtschaft/ernte-und-qualitaet/, accessed Feb. 2021

^b^FN3PT - Surfaces for seed certification from 2017 to 2019

^c^NAK National Seed Certification Service

^d^AHDB; Variety analysis · Potato Data Centre (ahdb.org.uk)

^e^FIWAP/PCA, 2021www.fiwap.be, annual enquiry FIWAP, PCA, INAGRO, CARAH

^1^For France and the Netherlands, no data are available on the Top-10 varieties combining the different types of potato production, only for seed

In France, the cropped area of around 200,000 ha is mainly dedicated to consumption potato production with a share of 77.3% (Table 5). Within consumption, around a third is for domestic fresh market and a third for food processing industry, and the remaining third is for exportation (https://agreste.agriculture.gouv.fr). Starch potato and seed productions represent each around 11% of the cropped areas (Table 5). France shares the NWEC-05 leadership with Germany with a share of consumption production of 25.8% (Table 6) and a slightly lower share cropped area of 25.1% (Table 5). French seed production is also significant, holding the second place in NWEC-05 after the Netherlands in terms of cropped area and production. No French data are available on the Top-10 varieties combining the different types of potato production; therefore, Table 7 presents the Top-10 varieties for seed potato production as average value for 2017, 2018 and 2019. Whilst a significant part of the seed production is dedicated to export (Table 12), as this is the case for the consumption variety Spunta, Table 7 clearly illustrates the share of cropped areas within the country for the different types of potato production which use more than 95% of certified seed tubers. Excluding Spunta, the five main varieties used in the country for food processing industry are identified (Fontane, Innovator, Challenger, Markies, Bintje), together with the two major varieties for starch production (Amyla and Kaptah Vandel), and two major varieties for fresh market (Agata, Monalisa). A wide diversity of other varieties for fresh market are cropped which have been more and more selected for fine skin finish for marketing after washing. A growing part of the table potatoes has been taken by salad potatoes registered in the specific French variety category called “Chair ferme” (Firm flesh) which accounts for almost 50 varieties at this date with the major ones being Charlotte and Amandine. Over the period 2017–2020, the survey conducted by the Interprofessional National Organisation (CNIPT) on the potatoes sold on the domestic fresh market shows that nine of the Top-10 varieties are from this category. On a secondary aspect, a growing part of the consumption potatoes has been produced since 2005 in the North of the country specifically for the Belgian processing factories producing French fries, which illustrates the huge pressure of the neighbouring Belgian potato industry. It explains the importance of this kind of variety in Table 7.

The Netherlands in several aspects is an exception to the average country in NWEC-05. It has the highest proportion of arable land dedicated to the potato crop (around 16%, Fig. 2) with 163,700 ha of potato, and the highest proportion of its area dedicated to seed potato production (26.2%), followed by the UK (Scotland) with 13%, so one country approaching almost half (43%) of the NWEC-05 seed production (Tables 5 and 6). About 27% of the potato crop is destined for starch production, quite comparable to Germany with around 20% resulting in both countries contributing about nearly 80% to the total NWEC-05 starch potato area (Tables 5 and 6). Country share for consumption potato is the lowest in NWEC-05 with about 50% of the potato arable land dedicated to this production, resulting in the lowest share within NWEC-05 for consumption potato production and cropped area (Tables 5 and 6).

No Dutch data are available on the Top-10 varieties combining the different types of potato production; therefore, Table 7 presents the Top-10 varieties for seed potato production as average value for 2017, 2018 and 2019, showing an equal distribution of varieties destined for French fries and fresh market production. Of the 1.48 million tonnes of seed potatoes produced (Table 7), 75% is exported all over the globe, especially Spunta to North Africa, and Fontane to NWEC-05 and neighbouring countries for seed supply to the expanding processing industry.

In the UK, the majority of the crops are grown for fresh consumption and processing (87%) (Table 5), and there is a significant seed sector with production predominantly in Scotland, with more limited seed tonnage produced in England and Northern Ireland. The Top-10 most widely grown varieties account for over 40% of the planted potato area (Table 7) and although Maris Piper is the most widely grown UK variety (11.8%), its dominance has declined as it accounted for 24% of the planted area in 1982. The dominance was in part because of resistance to *Globodera rostochiensis* and its suitability for fresh, processing and chips (French fries) shops trade. However, Maris Piper has been replaced by a range of other varieties with specific end uses, including Markies for French fries and chips shops. Innovator and Royal have also been used increasingly for French fry processing (Table 7). The shift to newer varieties continues and in 2020 Taurus overtook Lady Rosetta as the most popular crisping variety. A wide range of varieties are grown for the specialist UK pre-pack market (including Maris Peer and Charlotte) and as bakers (Melody and Nectar). The popularity of Maris Peer is also decreasing and competition is coming from newer varieties such as Jazzy. Seed potatoes are produced for both the UK market and export globally and the two main varieties grown in Scotland (2020) are Maris Piper and Hermes.

In Belgium, the major part of the cropped area is dedicated to consumption potato, and mainly for the processing industry, representing nearly 98% of the potato areas, as only quite a small part (2.4%) of the potato cropped area is dedicated to seed production (Table 5). No hectares are dedicated to starch potato production. Within consumption, as illustrated within the Top-10 cropped varieties (Table 7), most of the varieties (at least 83.4%) are cropped for food processing as French fries or crisps (chips) confirming the huge driving influence of the local processing industry in this country. In the last decade, variety Fontane showed a dramatic increase in cropped areas from 5600 ha in 2011 to 39,000 ha in 2019, whilst the historical variety Bintje decreased from 40,000 ha in 2011 to 9700 ha in 2019 (FIWAP/PCA, 2021). Innovator, Challenger and Markies are the other main varieties cropped in Belgium for processed French fries, VR808 and Lady Claire being the most cropped for processed crisps. Potato cropped areas for the fresh market are very low in Belgium rating with a maximum around 10% of the consumption production. Such a situation is clearly contrasting with Germany, France, the Netherlands and even the UK that show a larger share between consumption, starch and seed potato production. In these four countries, the variety share over the Top-10 ranges from 45 to 35% of the cropped area.

**Fig. 8:**
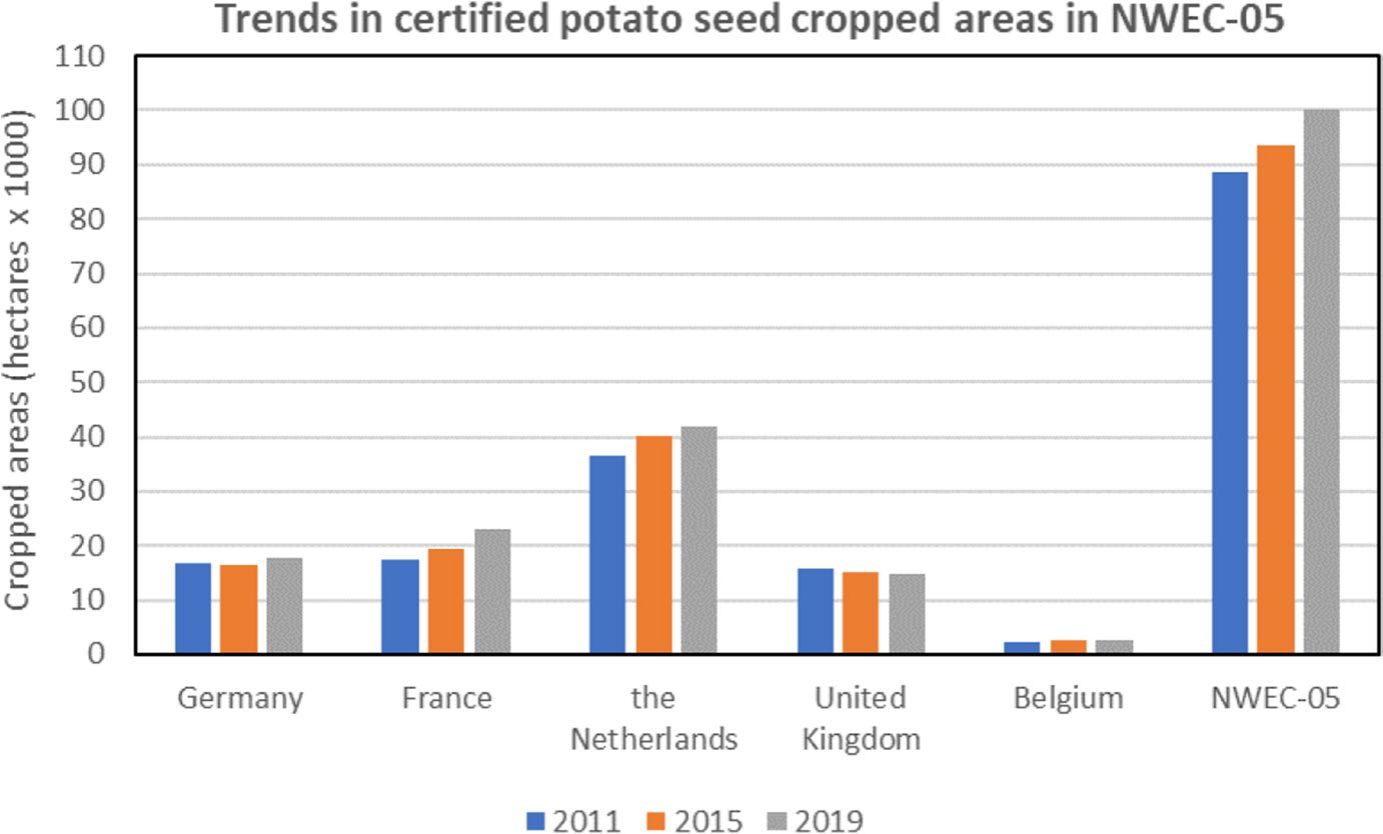
Certified potato seed cropped areas in 2011, 2015 and 2019 in each of the NWEC-05 under conventional agriculture. Sources: FIWAP/PCA, 2021, compilation of data collected from SPW-ALT (Belgium), NAK (the Netherlands), FN3PT (France), BSA (Germany), SASA (Scotland; for United Kingdom: data extrapolated from Scotland data that represent regularly around 70% of the United Kingdom certified seed production)

Specifically for potato certified seed production, more than 100,000 ha are currently cropped in NWEC-05 (Table 5). According to FIWAP/PCA (2021), over the last 10 years, the area increased from around 88,700 ha in 2011 to 100,000 ha in 2019 (Fig. 8). Mainly the Netherlands and France contributed to this increase according to their leading position for seed production, whilst Germany, the UK and Belgium maintained quite similar levels. The main varieties (i.e. with more than 800 ha) that are produced for seed in NWEC-05 are currently: Fontane, Innovator, Agria, Markies and Challenger for French fries processing; Hermes, Lady Claire and Lady Rosetta for crisps; Agata, Cara, Colomba, Monalisa, Belana and Jelly for fresh market; Spunta and Désirée for seed export outside NWEC-05 (FIWAP/PCA, 2021).

#### Organic Potato Production

Regarding organic potato cropped areas, the NWEC-05 represents 51.1% within EU-28, but the share organic/total is only 1.9%, similar to the EU-28 (Table 8). Germany is currently the most developed country in NWEC-05 for organic potato cropped area with a share organic/total of 3.6% within the country (Table 8). Germany is also number one in terms of share of organic potato area of 29.6% within EU-28 (Table 8), as the most other developed EU-28 countries for organic potato area are Austria, Denmark and Sweden with values of respectively 13.3%, 6.7% and 5.7% (Eurostat 2020). Considering the share of holdings producing organic potato with the total holdings producing potato in 2016 in NWEC-05 (Table 8), values range from 0.7% in the UK to the highest value in Germany (9.7%), with a mean value of 7.1% for NWEC-05. However, in EU-28, Austria and Sweden with a share of respectively 20.4% and 13.1% in 2016 are confirmed as the leading countries for organic potato production (Eurostat 2020). Other European countries with significantly developed organic potato production have share values of 10.1% for Denmark, 9.9% for Estonia, 9.1% for Luxemburg, 7.4% for Latvia and 7% for Finland.

**Table 8:** Organic potato cropped areas and holdings producing organic potato in NWEC-05 and EU-28

Country / Region	Organic potato cropped area^1^ (average 2017–2019)			Holdings producing organic potato (year 2016)^2^	
	Area	Share organic/total^3^ within the country/region	Share of EU-28 total	number	Share organic/total^4^ within the country/region
	ha	%	%		%
Germany	9345	3.6	29.6	2790	9.7
France	3370	1.5	10.6	2100	8.5
The Netherlands	1685	1.1	5.3	200	2.1
United Kingdom	1234	0.9	3.9	60	0.7
Belgium	845	0.09	2.7	130	1.0
**NWEC-05**	**16,479**	**1.9**	**51.1**	**5280**	**7.1**
EU-28	31,771	1.8	100	19,990	1.3

^1^Eurostat 2020: Eurostat (online data code: org_cropar) accessed 14 Jan 2021

^2^Eurostat 2020: Eurostat (online data code: ef_lac_rootcrop) accessed 7 Dec 2020

^3^Total potato cropped areas values from Table 3

^4^Total holdings producing organic potato values from Table 1

According to Regulation (EU) 2018/848 of the European Parliament and of the Council of 30 May 2018 on organic production and labelling of organic products and repealing Council Regulation (EC) No 834/2007 (http://data.europa.eu/eli/reg/2018/848/oj), organic potato production practices in NWEC-05 are based on the principle that no synthetic chemical compounds are applied. No compounds produced in factories are to be used but materials of organic plant origin refined or extracted in factories such as organic insecticides are often allowed. The manure and compost also have to come from animal farms that apply the organic principles. The seed potato should also be from an organic farming system. If a conventional farmer wants to convert to organic farming and wants to be certified, it is necessary to follow the organic rules for two or three years according to EU (regulation (EU) 2018/848 on organic production), country or association rules (e.g. Demeter, Bioland, Soil Association). There are some differences among countries regarding rules and regulations occasionally leading to discussions on level playing fields such as the use of copper in copper deficient fields that may also act as a fungicide.

### Economic and Trade Aspects of Potato Production in NWEC-05

In NWEC-05, potato is produced and valorized as an important cash crop for the producer and the whole potato value-chain. However, due to the absence of an organized common market and regulations systems at European level, the production is driven by supply and demand within the potato value-chain at national and international scales. Consequently, volatile price formation is the rule that results in huge variation in producer price that may mean potato producer incomes do not cover production costs, including storage costs for long periods from harvest in September–October to June–July in the following year. Research for sustainable ways of potato valorization is therefore an important issue for the NWEC-05. Together with proximal national/regional use, export of fresh potato, frozen processed potato products and potato seed intra and extra Europe were and are still current significant ways of valorization.

The following subsections develop some important aspects of the economy of conventional potato production in NWEC-05 that require thoughtful consideration for its sustainable development in the coming decades. Such aspects are holding potato production costs, highly variable annual production values due to producer potato basic price volatility and induced speculation and export as a way of production valorization. Some economical aspects of organic potato production in NWEC-05 are also developed.

#### Conventional Potato Production Costs (Consumption and Seed)

The evaluation of the potato production costs at holding level is not easy because it is related to a large number of factors whose costs may vary a lot between holdings (e.g., potato seed costs linked to the cropped variety). Therefore, each holding needs to establish its own specific annual production costs. However, it is useful to have a basic approach to the production costs for the NWEC-05 that could be compared to potato price per tonne, considering the costs expressed in euros per tonne produced based on the yield per ha. Table 9 summarizes for each of the NWEC-05, and for a reference variety or main use, the contribution of main standard components of production costs expressed in euros per hectare of potato produced on the field including land rental, which is a current component of potato production costs in NWEC-05, mainly Belgium and the Netherlands. The evaluation does not include either transport costs from the field to the on-farm storage facilities, or supplemental labour other than the producer, or any other costs than those mentioned in the table. On-farm conditioning and storage costs are not included.

**Table 9:** Estimated basic conventional potato production costs (in euros per hectare) for main components at field scale within NWEC-05 for a reference variety or main use in each country. Average data 2017–2019

Reference variety or main use	Germany^1^	France^2^	The Netherlands^3^	United Kingdom^4^	Belgium^5^	Average value for NWEC-05	Share of total cost
	*Belana*	*For processing use*	*Agria*	*For fresh market*	*Fontane*		*%*
**Components**							
Seed	1580	900	1660	906	1220	***1253***	**23.5**
Pesticides	453	500	620	674	740	***597***	**11.2**
Agricultural works contractors	419	770	630	252	570	***528***	9.9
Depreciation of equipment	342	760	400	671	400	***515***	9.7
Maintenance on equipment	217	100	520	446	100	***277***	5.2
Interest on capital	138	30	480	103	175	***185***	3.5
Renting land	430	460	1250	565	1250	***791***	**14.9**
Other variable or fixed costs	525	485	920	1470	300	***740***	13.9
**Total costs (€ per ha)**	**4518**	**4355**	**7020**	**5532**	**5185**	***5322***	**100**
Yield per ha (t)	41.2	49.9	61.0	45.4	46.1	*48.7*	
**Total costs (€ per tonne)**	**110**	**89**	**115**	**121**	**110**	***109***	

^1^100% certified seed; https://www.stmelf.bayern.de/idb/speisekartoffeln.html, accessed Jan. 2021; https://tlllr.thueringen.de/fileadmin/TLLLR/Service/Publikationen/BW_Richtwerte/2019_10_09_BRW_Speisekartoffeln.pdf, accessed Jan. 2021; https://www.bmel-statistik.de/archiv/statistisches-jahrbuch/, accessed Feb. 2021

^2^Arvalis Institut du Végétal, France: average seasons 2017-2018-2019 on type farm specialized on potato for processing cropped with irrigation in Northern France

^3^KWIN PPO Lelystad, the Netherlands, Agrarische kengetallen

^4^AHDB, Great Britain, Cost of Production for fresh market potatoes 2017–2019

^5^FIWAP (Gembloux) and Proef Centrum voor Aardappelteelt (PCA, Kruishoutem) Belgium

Current average production costs (excluding transport from field to storage facilities, on-farm conditioning and storage costs) is 5322 € ha^−1^ for NWEC-05 region, ranging from 4518 € ha^−1^ in Germany to 7020 € ha^−1^ in the Netherlands (Table 9). The national data are not fully comparable as figures are from different sources so there are systematic differences mainly due to variety and production type. However, it gives an overview of cost per ha for NWEC-05. Considering an average yield around 49 t ha^−1^, and average total costs of 5322 € ha^−1^ (Table 9), a basic potato price of about 109 € per tonne is required to balance the production costs in NWEC-05 production conditions. Including other variable costs components such as costs for transport, on-farm conditioning and storage for a long period can lead to a basic price ranging from 140 to 160 € per tonne. Considering the average share of the different production costs components (Table 9), the seed costs (24% of total costs), renting land (16% of total costs) and pesticides (11% of total costs) represent all together nearly half of the production costs. Other specific components including fertilizer costs represent around 35% of the costs. Mitigating seed, pesticides and land rental costs appear to be first options for farmers to substantially reduce total production costs.

#### Conventional Potato Production Value and Potato Producer Basic Price

Table 10 supplies the global potato output value in the NWEC-05 at producer basic potato price as average value for the period 2017–2018 (Eurostat 2020). Share of each country at NWEC-05 and EU-28 levels are given, together with the comparison of global potato output value share to global total agriculture output value. Germany and France are the leaders in potato value with respective shares of 25.0% and 37.5% of NWEC-05. The Netherlands has a medium position, and the UK and Belgium have the lowest contributions. Remarkably, the NWEC-05 together represents 64% of the EU-28 potato value output, and 4.1% of the total agricultural output. It is to be noted that within NWEC-05 the Netherlands and Belgium have the highest national potato shares of total agriculture. In Belgium that is likely to be related to the huge expansion of the proportion of the potato crop share of arable land over the last two decades (Fig. 2).

**Table 10:** Basic potato output production value at producer potato basic price (average value for 2017–2018) based on free market potato prices

Country/Region	Potato output value	Share of NWCE-05 total	Share of EU-28 total	Total agricultural output value	Potato share of total agricultural output
	x 000 000 euros	%	%	x 000 000 euros	%
Germany	1950.8	**25.0**	*16.1*	53,439.7	3.7
France	2923.8	**37.5**	*24.0*	73,426.8	4.0
The Netherlands	1539.5	**19.7**	*12.7*	27,733.9	5.6
United Kingdom	891.0	**11.4**	*7.3*	28,272.4	3.2
Belgium	491.9	**6.4**	*4.0*	8258.2	6.0
**NWEC-05**	**7797.0**	**100**	***64.0***	**191,131.0**	**4.1**
EU-28 (excluding NWEC-05)	4387.4	-	36.0	227,284.9	1.9
EU-28	12,184.4	-	100	418,415.9	2.9

Source: EUROSTAT 2020 (Eurostat, online data code: aact_eaa01), last update 2020 Dec 16, http://appsso.eurostat.ec.europa.eu/nui/submitViewTableAction.do

**Fig. 9:**
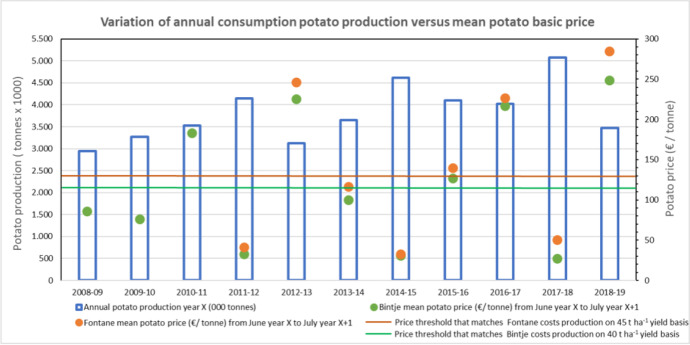
Evolution of annual production of consumption potato and annual mean producer potato basic price for two main varieties (Fontane and Bintje) in Belgium over the period 2008–2019. Source: FIWAP/PCA Belgium (https://fiwap.be/article/stocks-belges-au-1er-fevrier-2021/)

Fig. 9 provides an example of high volatility of producer basic potato prices on the free market using data for two varieties, Bintje and Fontane, of consumption potatoes in Belgium over the 2008 to 2019 period. The producer potato basic prices are mean annual value from June of the production year to July of the subsequent year. For both varieties, annual mean prices have fluctuated from 30 € per tonne up to more than 250 € per tonne, meaning an 8- to 9-fold variation. As already described, such huge price variations are mainly related to the annual variation of potato production (and to a lesser extent the annual tuber quality for processing or fresh market), not just in Belgium but also in the other neighbouring NWEC-05, and finally to total European production volume. This impacts on the tuber supply to match mainly the processing industry demand in NWEC-05, and the availability of production for the fresh market traded intra and extra NWEC-05.

Such a high price volatility also illustrates the huge vulnerability of the NWEC-05 potato sector on a short-term basis. Considering the Belgian case, and on realistic 40 and 45 t ha^−1^ yield basis respectively for variety Bintje and Fontane over the considered period, the production value per ha to match the production costs of 5185 € (Table 9) requires producer basic prices of at least 130 € and 115 € per tonne for Bintje and Fontane, respectively, as illustrated in Fig. 9. Over the 2008–2019 period, such prices only appeared for five of the 11 years for Bintje, and for four of the 8 years for Fontane, indicating that the economic sustainability of the potato production requires a multi-year approach. This means producers have to be highly professional, with a well-equipped holding for potato production and significant investments in specific machinery and ideally own or have access to nearby storage facilities.

It is important to note that the Eurostats production output values in Table 10 are based on the free-market price and therefore could erroneously estimate the value. In order to approach the real value of potato production, it is necessary to integrate the valorization under contract. Considering the variety Fontane in Belgium over the 2017 to 2019 harvests (Table 9, FIWAP/PCA, 2021), the average marketable yield is 46.1 t ha^−1^ (varying from 37.7 t ha^−1^ in 2018 to 55.7 t ha^−1^ in 2017). The average selling price (combining the free market and contracts and taking into account the volumes sold over four periods during the year on the basis of stock surveys) is 124 € per tonne (varying from 91 € per tonne in 2017 to 160 € per tonne in 2018). The average gross revenue is therefore 5537 € ha^−1^ (varying from 5083 € ha^−1^ in 2017 to 6194 € ha^−1^ in 2018). This example illustrates the large variability of the yield, but also the buffer effect of the contracts on the average sale value. Indeed, during the considered period, the average contracted sales value fluctuated by 19% from 113.70 € per tonne for the 2018 harvest to 135.70 € per tonne for the 2019 harvest, whilst the average free market value fluctuated by 83% from 48.40 € per tonne for the 2017 harvest to 286.1 € per tonne for the 2018 harvest.

#### Valorization of NWEC-05 Conventional Potato Production Through Exportation

A significant part of the potatoes produced in NWEC-05 is exported either intra EU-28 or extra EU-28. The potato volumes exported from each country of the NWEC-05 as potato for food consumption (for fresh market or as frozen processed potato products, including early potato production), as potato for starch production or as potato seeds are detailed in Table 11, together with their share at NWEC-05 and EU-28 levels, as average annual values for the period 2017–2019. The exported part for each of the main types of potato production is illustrated in Table 12 for the same period.

**Table 11:** Average annual exportations of NWEC-05 potato production (including intra and extra EU-28 exportations) for the period 2017–2019

Country / Region	Consumption^1^			Starch			Certified Seed		
	Export	Share of NWEC-05	*Share of EU-28*	Export	Share of NWEC-05	*Share of EU-28*	Export	Share of NWEC-05	*Share of EU-28*
	x 000 tonnes	%	***%***	x 000 tonnes	%	***%***	x 000 tonnes	%	***%***
Germany	1788.4	**30.7**	*26.7*	53.4	32.2	*29.8*	92.8	6.5	*6.0*
France	1946.2	**33.4**	*29.1*	87.5	52.8	*48.9*	201.2	14.1	*13.0*
The Netherlands	1011.4	17.3	*13.5*	15.3	9.2	*8.5*	947.4	**66.3**	*61.1*
United Kingdom	181.5	3.1	*2.7*	0.8	0.6	*0.4*	97.8	6.8	*6.3*
Belgium	901.9	15.5	*13.5*	8.6	5.2	*4.8*	90.0	6.3	*5.8*
**NWEC-05**	**5829.4**	100	***87.1***	**165.6**	100	***92.3***	**1429.2**	100	***92.2***
EU-28	6692.6			179.0			1549.9		

^1^Consumption potato for fresh market or as frozen processed potato products, including early potato production. Source: EUROSTAT 2020, Eurostat (online data code: DS-016890), accessed on 29 Dec 2020

**Table 12:** Exported part of the NWEC-05 potato productions (including intra and extra EU-28 exportations) for the period 2017–2019

Country/Region	Consumption^1^			Starch			Certified Seed		
	Production^2^	Export^3^	% exp	Production^2^	Export^3^	% exp.	Production^2^	Export^3^	% exp
	x 000 tonnes			x 000 tonnes			x 000 tonnes		
Germany	6523.1	1788.4	*27.4*	2080.0	53.4	*2.6*	548.0	92.8	16.9
France	6543.5	1946.2	*29.7*	1011.2	87.5	*8.7*	768.0	201.2	**26.2**
The Netherlands	3599.7	1011.4	*28.1*	1713.0	15.3	*1.0*	1480.1	947.4	**64.0**
United Kingdom	4920.2	181.5	*3.7*	0	*[0.8]*	*0*	589.8	97.8	16.6
Belgium	3762.7	901.9	*24.0*	0	*[8.6]*	*0*	67.2	*[90.0]*^4^	*[100]*^4^
**NWEC-05**	**25,349.2**	**5829.4**	***23.0***	**4804.2**	**156.2**	***3.3***	**3412.4**	**1429.4**	**41.9**

^1^Consumption potato for fresh market or as frozen processed potato products, including early potato production

^2^Values from Table 6

^3^Values from Table 11

^4^See explanation of the values in text

According to Table 11, NWEC-05 represented a huge share of around 90% of the total EU-28 potato production exported intra or extra EU-28, meaning that the other EU-28 members have quite reduced potato export activity. With a share of around 30–35% of NWEC-05, Germany and France are both leaders for export activity (mainly intra EU-28) of their production of consumption potato (fresh market and frozen potato products), representing 30% of its production for France and 27% for Germany (Table 12). The Netherlands (with 75% intra EU-28) and Belgium (quite exclusively intra EU-28) with respectively 28% and 24% of their consumption production dedicated to export (Table 12), represent each one a lower share of around 14% within NWEC-5 (Table 11). The UK has a reduced export activity for potato consumption but has the highest level of exports in the NWEC-05 market for potato crisps. A poor export activity in NWEC-05 is observed for potatoes dedicated to starch production. Concerning export of potato seed, the Netherlands (with 60% intra EU-28) dominates with a share of around 66% of NWEC-05 and EU-28, representing around 65% of its potato seed production. France is also an important NWEC-05 actor for seed export with a share of 14%, representing 26% of its potato seeds production and around 50:50 intra and extra EU-28. To a lesser extent, Germany, UK and Belgium are similar contributors as NWEC-05 potato seed exporters. It must be noted that for Belgium (Pierre Lebrun, personal communication 2021), the higher seed export compared to seed production in Table 12 is explained by taking into account that some production of neighbouring countries (Luxemburg, Germany) is commercialized and exported by Belgian traders. Belgian processing factories also deliver seed potatoes to foreign potato producers, then considered as seed export, in connection with potato purchases contract. Besides exporting their own potato production, NWEC-05 and mainly the processing industry in the Netherlands and Belgium, also exported frozen processed potato production from NWEC-05 to Europe, but mostly to countries in Asia, the Middle East and Latin America. These countries show increasing potato consumption that is the result of higher disposable incomes, a growing middle class, urbanization and the expansion of fast-food outlets in these countries.

#### Economical Aspects of Organic Potato Production in NWEC-05

Organic potato production is usually more expensive per tonne produced than conventional crop as reflected by the higher consumer prices. Potato seed prices are much higher and yields are lower. The fertilizer input is sometimes lower (when mainly based on farm manure) but can also be very expensive when mainly based on processed organic fertilizers. Fungicide and insecticide use (with organic related products) are less expensive. Late blight used to be a huge issue in organic potato production with global productions varying heavily depending on late blight attacks. For some heavy late blight years such as 2012, 2014 and 2016 (and even 2021), many fields were heavily affected and destroyed by *Phytophthora infestans* (from Aardappelwereld, 2016)*.* However, the increasing use of the so-called robust varieties which are late blight tolerant or even resistant (Queisen 2020) will hopefully help the organic potato sector to produce more potatoes per ha and on a more regular level.

Producer prices for organic potatoes are usually more stable and higher than conventional ones. German organic producer basic prices for instance have fluctuated between 400 and 700 €/tonne over 2015 to 2018 (AMI GmbH). Tuber rejection levels are usually much higher, varying from 20 to more than 50%, which is significantly more than for conventional production where rejection values range from 5 to 20%. Storage of organic potatoes does not take place using synthetically produced products but plant-derived products from mint and carvone oil and limonene (orange extract) are used (Frazier et al*.* 2014) depending on national regulations. Traditionally, organic growers invested early in refrigerated cooling to suppress sprouting with the help of long dormancy varieties.

### Environmental Footprint of the Potato Production in NWEC-05

The environmental footprint of the potato crop is considerable. The crop cannot be grown with low or no tillage and a lot of soil needs to be moved and loosened during the operations of ploughing, planting, hilling and harvesting, responsible for much energy at the cost of CO_2_ emission. A major share of energy is embedded in nitrogen fertilizer, irrigation and storage. Even where decision support systems (DSS) have been adopted over the past 10 years to protect the crop against late blight and tend to reduce the number of fungicide applications, these sprays also add up. So compared with cereals where low tillage, few sprays, no soil movements, no irrigation and storage at ambient temperatures suffice, potato has some environmental issues.

The Cool-Farm-Tool potato developed in the Netherlands by Haverkort and Hillier (2011) quantified the energy input of four potato production cropping systems (conventional consumption, organic consumption, conventional seed, conventional starch) as CO_2_ embedded therein (Table 13). Fertilization has two components: fertilizer production in the factory and emission of mainly methane from the soil. Production of manure is also at the cost of CO_2_ but this is associated with the animal industry. Transport of it and compost to the farm is attributed to the potato crop. To produce one tonne of conventional table potatoes in the Netherlands costs 80 kg of CO_2_. Organic table potatoes produce more CO_2_, none in chemicals but more in manure/compost transport and emissions and in cooling the crop after harvest. Both seed and organic have low yields, therefore their footprint is relatively high. The figures for other countries will differ somewhat with more irrigation in France and less use of manure there but Table 13 represents a clear picture of the footprint of potato and how to calculate it. The footprint of wheat is much higher per tonne on a fresh weight basis, about three times as high. However, if we assume that potatoes contain 78% water and wheat grains only 15%, on a dry weight basis the potato footprint is some 30% higher than that of cereals. This is because potatoes are hardly irrigated, and for cereals lower tillage and less soil movements occur than with potatoes, less crop protection chemicals are applied and no ventilation nor refrigeration is needed to store cereals.

**Table 13:** Environmental cost of producing 1 t of tubers for four potato production cropping systems (conventional consumption, organic consumption, conventional seed, conventional starch) expressed as kg CO_2_ embedded in material or energy costing operations (field cultural practices, irrigation and storage) (from Haverkort and Hillier 2011)

Production Factor	Potato production systems			
	Conventional consumption	Organic consumption	Conventional seed	Conventional starch
Seed	4	7	15	3
Fertilizer production	25	0	40	12
Fertilizer emissions	25	27	18	32
Biocides	5	0	16	10
Field operations	7	26	11	11
Irrigation	1	2	0	0
Storage	10	23	15	2
Manure/compost transport	3	20	2	17
**Total**	**80**	**105**	**117**	**87**

## Conventional potato production practices and storage in NWEC-05

Over the last decade, an increasing level of potato production in NWEC-05 has been driven, mainly by the frozen processing industry, to maintain a year-round supply of high-quality tubers. This means that the production potato sector in NWEC-05 invested considerably in knowledge, mechanization, storage and irrigation to achieve increasing production. Similarly, the sector of seed potato production has invested in the development of technologies for high quality seed.

The next subsections present an overview of the different potato production methods and techniques applied in NWEC-05, with a focus on country-specific approach or equipment where required.

### Soil Preparation, Planting, Ridging and Herbicides Application

First, to prevent the build-up of weeds and pathogens in the soil, it is recommended farmers avoid growing potatoes on the same land from year to year. So, they generally grow potatoes typically in rotations of three or more years when arable land is available, alternating with other crops, such as maize, cereals, sugar beet, rape seed and vegetables. However, few farmers still unfortunately produce potato in a 2-year rotation, predominantly in case of starch potatoes.

Intensive ground preparation is required. To reach suitable conditions for planting, the soil with a suitable level of moisture is generally ploughed once and then harrowed usually at least twice until completely free of weed roots and the soil is friable, well drained and well aerated. In the UK, stones and clods separation is widely adopted, although in recent years shallower, less intensive cultivation operations are being implemented with introduction of winter cover crops.

Planting is ideally operated using about two tonnes per hectare of good quality commercial certified seed potatoes, that are disease-free, ideally well sprouted and with a planting density in row depending on the size of the seeds tubers and expected stem numbers. Farm saved seed use is allowed theoretically with multiplication fees to the variety right holder (EuGH C-242/14), but certified seeds is highly recommended whilst expected profits must offset their higher cost. Crops are grown on ridges that are in most cases earthed up once at the mean time of planting and generally four rows at a time. However, on clay soils (especially the Netherlands) planting and hilling are two separate operations. Surface soil of the ridges must be as loose and fine as possible to allow a homogenous distribution of the herbicides that are ideally applied 10 to 15 days after planting and before crop emergence. Post-emergence herbicides applications are usual in case of bad weather conditions that prevent pre-emergence application. For seed production, planting in a three rows bed is also used to promote the production of numerous tubers of small and medium size at high level of plant density. The use of precision planting using a tractor mounted geolocalized positioning system (GPS) for planting is developing in the NWEC-05 and modification of planting density according to soil characteristics also starts to develop (Kempenaar et al. 2017).

### Fertilization

Chemical NPK fertilizers are still mostly used in conventional potato production, using fertilizer sprayers or spreaders of high capacity. It is recommended and generally respected that crop NPK fertilization requirements need to be correctly estimated according to the expected yield, the potential of the variety and the intended use of the harvested crop. For N, it is mainly based on a provisional balance sheet approach at field scale. Splitting or within-field modification of the N applications became available and is encouraged through access to platforms and application maps using within field geolocalization and data collected from optical sensors embedded on satellites or on sprayer or tractors, to better match the N fertilizer supply with the variable crop N needs in space and time (Goffart et al. 2008; Goffart et al. 2017; Kempenaar et al. 2017; Janssens et al. 2020). Regarding the application of the European Nitrate Directives (91/676/EEC) reducing nitrate pollution from agricultural land, Fertilization Ordinances have been enacted in the individual NWEC-05. Furthermore, some European countries have also restricted P_2_O_5_ under different legislations (e.g. EU Water Framework Directive (Directive 2000/60/EC), Fertilization Ordinances) (Amery and Schoumans 2014).

### Irrigation

Between 40 to 60% of the potato-cropped area is irrigated in Germany, France, the Netherlands and the UK, whilst Belgium has only around 5%. Most of the irrigation systems and equipment are well developed and diversified. Professional extension and agronomy services are active for in-season irrigation recommendations to producers with the help of adapted DSS or direct soil probes.

Due to climate change producing hotter and drier weather conditions in summer, water availability is sometimes scarce, and increasingly there are water restriction uses, mainly in France and the UK with regional management and to a lesser extent in the Netherlands and more recently in Belgium. In the Netherlands, irrigation from deep wells near the coast is problematic in most areas because of salinity. For seed potato, irrigation is forbidden because of risk of contamination with brown rot (*Ralstonia solanacearum*).

### Crop Protection

General precautions against pests and diseases are applied, such as crop rotation, use of tolerant or resistant varieties and use of healthy and certified seed tubers. Bacterial and viral diseases are controlled by regular monitoring (and when necessary, spraying) of their aphid vectors. Regarding the control of fungal diseases such as late blight, relevant and efficient DSS together with use of tolerant/resistant varieties are developed across all the potato cropped areas in the NWEC-05, with the objective to limit the use of fungicides (Haverkort et al. 2009). Indeed, late blight (*Phytophthora infestans*) in most situations still requires between 10 and 20 applications of fungicides over the growing season depending on the potential infection risks and inoculum pressure. Early blight (caused by *Alternaria spp*.) is also increasing and its control can require two or three fungicides applications. Sanitation, crop rotations and use of resistant potato varieties help control and prevent the spread of potato cyst nematodes (*Globodera* spp). However, problems with root knot nematodes (*Meloidogyne* spp.) or free-living nematodes (*Pratylenchus* spp., *Trichodorus* spp.) as virus vectors seem to increase (in the Netherlands and in Flanders-Belgium) with no resistant varieties currently available. Damage caused by the Colorado potato beetle (*Leptinotarsa decemlineata*), a major pest (except in the UK), still remains under control by destroying beetles, eggs and larvae that appear through targeted early application of insecticides. Precision geolocalized application of fungicides are in development over most of the NWEC-05 potato cropped areas, with the aim to optimize use through variable rate application based on biomass map and DSS (Kempenaar et al. 2017). The crop also copes with new emerging or re-emergent pests and diseases such as the appearance of the red spider mite (*Tetranychus urticae*) or the *Candidatus*-Liberibacter-psyllid plant complex (https://gd.eppo.int/taxon/LIBEPS/distribution/BE).

### Harvest

To facilitate harvesting, the potato foliage is destroyed at least 3 to 4 weeks before harvest when necessary. This foliage destruction also limits late blight tuber contamination and promotes tuber skin resistance before harvesting operations and so limits damage which would reduce the tuber ability for long-term storage. It is to be noted that this is not the case for early varieties which are harvested directly, with harvester machines equipped for flailing. Chemical or mechanical haulm killing, or a combination of both, is operated when the crop is assessed as mature for harvest, processing and/or storage. The quality of the harvested crops is crucial for the intended end-markets and the biochemical and physical tuber characteristics play a critical role (Storey 2007). Potatoes are mostly harvested using commercial potato harvesters that unearth the plant and shake or blow the soil from the tubers, and that limit bruising or other injury, which provide entry points for storage diseases and affect the tubers’ quality. The degree of mechanization for harvest is very high and costly in the NWEC-05.

### Storage

To avoid tuber deterioration and post-harvest losses of potatoes destined for fresh consumption or processing, tubers are stored in large facilities under controlled environmental conditions. They are kept in well-equipped large storage facilities generally at temperatures ranging from 2 to 10 °C, in a dark and well-ventilated environment with high relative humidity (85 to 98%). The holding temperature used depends on the intended end-use; with fresh market potatoes held at lower temperatures, of 2.5 to 4 °C often with refrigeration, whereas crops intended for processing are held at higher temperatures of 6 to 10 °C to avoid reducing sugar accumulation which will result in brown colored fries and crisps and higher levels of acrylamide in processed products (Foot et al. 2007). Stored tubers held above 4 °C are also usually treated with sprout suppressant to avoid sprout initiation and growth and the recent European non-renewal of chlorpropham (CIPC) as sprout suppressant has created a huge issue for the storage of potatoes in Europe (see later section). In recent years, Germany, France, the Netherlands and the UK have generally increased the proportion of good storage facilities with improved environmental control (including cooled stores) and pallet boxes; however, Belgium still remains with more basic storage facilities, whilst developing new facilities has accelerated.

Seed tubers on the other hand are held in low temperature refrigerated stores without the use of suppressants from autumn up to the next cropping season. Seed may then be moved to chitting sheds with diffused light to encourage development of vigorous strong sprouts for planting. One recent development is the use of temporary sprout suppressants, for some varieties, aiming to exploit higher storage temperature and increased tuberization.

## Current Issues and Challenges of Potato Production in NWEC-05 and Short to Mid-Term Solutions Proposal for Sustainable Production

Previous sections described and identified several current characteristics and specificities of the potato production in NWEC-05. However, the last decades development of potato production raises the question of its future ability to develop further in a sustainable way given the current environmental, economic, societal, political but also scientific constraints and opportunities. Issues and challenges that potato production will face need to be identified and formulated, and a SWOT analysis of the current potato production in NWEC-05 will help this approach. Solutions to answer current issues and challenges will then be proposed for a sustainable potato production in this region based on strengths and opportunities for the potato production.

### SWOT Analysis of Current Potato Production in NWEC-05

Table 14 summarizes a SWOT analysis of the current potato production in NWEC-05. It identifies the internal strengths and weaknesses of the potato production and sector in the region, and the opportunities and threats from external factors acting on the potato production, based on the descriptive approach of previous sections and on the expertise of the authors.

**Table 14:** SWOT analysis of current potato production in NWEC-05

**Strengths**	**Weaknesses**
Historically highly favourable soil and climate condition for potato crop growth, leading to the highest potato yields at world scale	Compared to unirrigated cereals, irrigated potato crops have a higher CO_2_ footprint on a dry matter yield basis.
Highly trained and / or experienced potato farmers and qualified agronomists	Use of high amounts of pesticides for disease/pest/weed control
Development of integrated supply chains from breeding and seed production to end market	Root system with low N-efficiency resulting in high N-inputs and risks on N-leaching
Huge expansion of processing industry, mainly for potato-based frozen products	Requirement for a high level of maintenance of the technology level applied
Availability and access to new high technologies for potato production, crop protection and storage	Soil ridging causes water and nutrient run-off and erosion on sloped fields
High level academic and public research to support new issues for the potato sector	High volatility in potato price formation versus increasing production costs
Presence of important and relevant potato support/extension services in the whole NWEC-05 territory for technical and economic recommendation	Lack of high level of public cooperation between countries for common solution to production issues
Existing international potato organizations such as Europatat (trade), EUPPA (processors) and NEPG (growers)	Economic issues between potato value chain stakeholders (need for clearer contractual policy)
Well-developed trade network for export of fresh and processed potato products	Brexit is making trade between the UK and the rest of Europe more difficult and expensive
The region is a real potato hub with its advantage of scale	Poor image of the potato sector in media and by general public
**Opportunities**	**Threats**
New breeding technologies enabling more rapid variety development	Decreasing potato consumption in Europe
Development of precision agriculture and remote sensing capabilities	New disease and pest issues (emerging, re-emerging)
Development of biocontrol products for potato disease, pest, weed and sprout control	Crop and tuber protection issues (active substance banning e.g., CIPC as sprout suppressant for storage, diquat for haulm killing, mancozeb for late blight control)
Development of mechanical alternative methods to chemical pesticides for disease/pest/weed/sprout control	Climate change and extreme weather events (increasing mean temperatures and increasing occurrence of in-season heat waves/ drought or heavy precipitation events and more challenging weather conditions (hotter) during the storage season (which in this case will lead to a more important use of cooling units (higher costs, higher CO_2_ footprint))
Increasing demand for organic potato production	Effects of intensified potato production on global soil fertility and soil health situation, (disease/pest pressure and evolution, nematodes, water run-off, soil erosion, soil compaction)
Potentiality in increased cooperation for different types of potato production within NWEC-05 and some others countries in NWE	Additional irrigation needs are required in some areas (F, D, UK) to maintain or reach higher yields
Climate change leads to longer growing seasons and higher crop growth rates and yields	Effect of COVID-19 pandemic on global potato production in NWEC-05 that will temporary negatively impact potato valorization at regional and international scale
Increasing short potato marketing channel	Irrational position of lobbies or marketing operators on cultivations practices (e.g. “No residues” campaigns vs Maximal residual limit (MRL) regulation)
Increasing demands for knowledge, seed tubers, potato products in developing countries	Excessive and uncontrolled development of potato processing (logistics, environment, stakeholders’ relations in the potato value-chain)

### Discussion on Issues, Challenges and Solutions Proposal

The SWOT analysis clearly states and summarizes the reasons for the development over centuries of potato crop production in the NWEC-05 region and mainly its still increasing development over the last two or three decades, whilst an overall decline was observed in Europe starting in 1960 (Devaux et al. 2020). Highly favourable soil and climate conditions, professional stakeholders within a performing integrated supply chain from breeding to market, expansion of processing industry, relevant research/support/extension services over the whole region together with a well-developed trade network intra and extra Europe are the main drivers of this success story. Potato production in NWEC-05 is therefore part of one of the most productive industrial agri-food systems worldwide. However, the sustainability of such development trends towards agricultural intensification to achieve more outputs per unit of land, is under debate, including for potato production, particularly considering agriculture’s environmental impact and footprint (Haverkort et al. 2013b). Technical, societal, economic and political issues and challenges are also part of the debate. Therefore, solutions for sustainable potato development in NWEC-05 in the next decades are urgently sought. To structure the identification of solutions, the issues and challenges identified through the SWOT analysis have been grouped into three main sections:

- Issues related to degradation of natural resources and to current and upcoming climatic context
- Issues related to the use and/or presence of pesticides and chemicals in production, storage and processing
- Challenges for efficiency and sustainability of the potato value-chain at national, regional and outside Europe levels.

#### Issues Related to Degradation of Natural Resources and to Current and Upcoming Climatic Context

Main environmental issues for the potato production in NWEC-05 focus on crop management factors such as soil fertility and health, and on water and fertilizers requirements.

The stable trend of potato yield over the two last decades and an apparent decrease over the recent years in NWEC-05, comparative to the significant yield increase in the rest of EU-28, is to be monitored in the coming years. The growing trend for intensive potato crop can lead to negative effects on soil fertility and soil health, which can eventually hamper yield potential in the mid to long term. The risk is particularly high for instance in Belgium, in a small territory (with an increasing share of potato on arable land area), and in the North of France, under the driving pressure of the rapidly expanding processing industry, requiring an increasing amount of potato and cropped areas. In the Netherlands, risk of impact on soil fertility and health is also to consider as it represents the highest share of potato on arable land area in NWEC-05. Such situations need to be analyzed and monitored as soil compaction or development of nematodes or other soil related pests and diseases are main contributing factors to soil fertility and soil health degradation. Short-term solutions should then be to respect a 4- or 5-year rotation at least, to apply rotation wide nematode management, to use lighter equipment and to respect traffic lane farming across the field and favourable soil access conditions for harvest.

Increasing occurrence of water run-off and soil erosion in fields historically not dedicated to potato crop because they have been too hilly or stony is becoming another important issue particularly due to increasing occurrence of extreme precipitation events. Adoption of contour farming, and the use of micro-dams in potato furrows to reduce erosion and runoff and minimize surface water contamination through pesticides (Olivier et al. 2014; Sittig et al. 2020) also appear as relevant short to mid-term solutions in sensitive fields and are disseminating.

Fertilizer use, mainly nitrogen, remains an issue due to the risk of N surplus responsible for water nitrate pollution and ammonia or nitrous oxide emission to the air contributing to greenhouse effect. For the short to mid-term, N recommendation at field scale combined with split and precision fertilizers application [as either management variable zones (MVZ) or variable rate application (VRA) based on remotely ground-based sensors or sensors embedded on UAVs or satellites] are now well-developed techniques to overcome excess application. The development of varieties with higher N and water use efficiencies will also help in mid to long-term allowing the application of lower N fertilizer rate. The respect of stricter (regional) regulation on fertilizer inputs (mainly nitrogen but also phosphorus) will also contribute to reduce environmental mineral fertilizers use issues.

Linked to climate change and increasing occurrence of extreme summer drought conditions, irrigation needs to cover either increasing water needs due to increasing evapotranspiration or lack of water during heat waves is becoming a huge issue in NWEC-05 for the potato crop, particularly in areas with problems of water availability and access. In the mid to long term, it will become challenging to set up varieties tolerant to drought showing higher water use efficiency, to develop drip irrigation, to avoid sandy soils and/or increase water logging capacity of soils, and finally to invest in reservoirs in the neighbouring fields and associated distribution networks.

#### Issues Related to the Use and/or Presence of Pesticides and Chemicals in Production, Storage and Processing

Under societal pressure links to human health hazards and negative effects on global biodiversity, the need to reduce chemical pesticides use whilst keeping efficient pest, disease and weed control is becoming a real technical issue and challenge for the future. Currently, the use of tolerant or resistant varieties to pests and diseases (e.g. late blight) is the main avenue to overcome this issue, and progresses are going on with cisgenic approaches (Haverkort et al. 2016) and NBTs (New Breeding Technologies) (Jansky and Spooner 2018), whilst their use in the European Union remains a huge challenge as it encountered political banning. In the United Kingdom, a public consultation has opened post-Brexit on adoption of cisgenic technologies as it is no longer a requirement to meet EU regulations.

Increased research on biopesticides and biostimulants combined with cropping of tolerant varieties to diseases will also be part of a solution. In the short to mid-term, such alternatives to chemical pesticides are urgently required due to rapid and increasing banning at European Union level of numerous chemical pesticide active substances used for pest, disease, weed control and haulm destruction, all of which have been used as efficient and cost-effective solutions for decades. Mechanical techniques for haulm destruction, potentially combined with chemical application of haulm killing substances, is fortunately quickly developing. The supply of site-specific amounts of chemical foliage desiccant based on the amount of green biomass measured in the field is now possible using embedded optical sensors on tractors or sprayers (van Evert et al. 2012; Kempenaar et al. 2017). Consequently, the amount of chemicals for haulm destruction is reduced up to 50% and is useful in the context of the current and future bans of some foliage desiccants in Europe (e.g. diquat). Improvement of new mechanical or electrical technologies could also help in the near future to solve this important technical step before harvesting in industrial production.

Due to climate change, occurrence and increasing pressure of pests and diseases, together with the (re-) emergence of new pests and diseases, will likely require a high and increasing number of chemical pesticides or alternative solutions. Globally, as stated by Kroschel et al. (2020), many insect pests of potato will respond to climate change by expanding their geographical range of distribution and increasing population densities will lead to greater crop and post-harvest losses, also in temperate regions. In the mid- to long term, modelling of the response of potato pest populations to global warming will be required to help predict potential changes in pest distribution and severity in order to support potato growers in the adaptation of their pest management strategies.

Another recent but major issue is the EC banning as of October 2020 of chlorpropham (CIPC) sprout suppressant for potato storage. It constitutes a major technical and economic challenge for the European potato sector to overcome this new situation, due to the high cost and limited knowledge of alternative solutions, and the historical contamination of storage infrastructure and handling equipment. This substance has been used since 1959 and has enabled the supply of markets (fresh and processing) for almost 12 months a year whilst controlling the quality of tubers, and has thus strongly contributed to the development of the European processing industry over the past 25 years.

The first main issue of concern is that the disappearance of CIPC will result in the end of the treatment of tubers by powdering, spraying and fogging as they were currently applied to storage. Alternative products exist, such as mint oil, ethylene, orange oil and di-methyl-naphthalene (DMN) approved in some countries in Europe, or 3 decen-2-one approved only in North America, all of which (except for pre-harvest maleic hydrazide) are applied exclusively by nebulization or by gaseous diffusion form. However, many of current storage facilities are not fully equipped for this technology across Europe. In addition, knowledge is lacking with these alternatives in terms of dose, period of application, ventilation schemes, combination of active ingredients, monitoring of efficacy and impact on the quality of tubers. In the short to mid-term, new research effort is therefore necessary to better promote these alternatives. In the current scenario, the additional costs may be borne by producers, as the issue of competitiveness of European finished products on world markets (mainly French fries) prevents a full pass-on of the additional costs to the end consumer. Complementary solutions are based on integration of technologies, more cooling, varieties low in reducing sugars and low temperature tolerance and vacuum frying in factories.

The second main issue is the uncertainty created by the historical contamination of storage buildings and potato handling equipment: the CIPC crystals encrusted in the materials (concrete in sheds, wood in pallet boxes, rubbers in conveyor belts, etc.) volatilize and CIPC is released and may deposit on potatoes now stored without CIPC. At the end of the legal period of use of CIPC, the maximum residual limit (MRL) in tubers should have been reduced theoretically to LOQ (analytical quantification limit), which would have represented an insurmountable challenge for producers because traces of CIPC can persist for up to 15 years after application, and no effective procedure for complete cleaning installations is known to date. However, efforts of the stakeholders of European Potato Value Chain have led to the establishment of a temporary MRL at 0.4 mg/kg for CIPC starting in September 2021 but for an undetermined period.

A last technical issue, linked to processed potato quality, is the presence of acrylamide in fried potato (processed French fries and potato crisps through temperature higher than 100 °C) that has been a matter of debate since its finding in 2002 (Tareke et al. 2002). It is classified as probably carcinogenic in humans, with significant toxicological effects. Acrylamide formation results from the Maillard reaction (Mottram et al. 2002) and has reducing sugars glucose and fructose, and asparagine as precursors. In the short to mid-term, there are two ways recognized to mitigate acrylamide formation: either by removing its precursors from raw potatoes through selecting relevant potato varieties, fertilizer use and storage conditions; or by applying processing methods including final preparation to inhibit or reduce the intensity of the Maillard reaction (Foot et al. 2007). Research efforts still need to be developed in the near-future in this context. However, it remains a huge challenge for the processing industry as tuber reducing sugars are also important to reach desirable sensorial properties in the final products such as flavour, odour, colour, texture and taste of French fries or crisps.

#### Challenges for Efficiency and Sustainability of the Potato Value-Chain at National, Regional and Outside Europe Levels

Cost-effective production of frozen processed potato products and crisps requires a year-round supply of high-quality potato tubers. It always requires higher yield and expansion of the cropped areas. It induces pressure on land accessibility and on land rental costs, such is the case particularly in Belgium and the Netherlands. Potato intensification in the same region also increases environmental issues and need for potato transport on longer distances to destination factories. The development of potato processing must respect local environmental, urban planning, social and economic regulations. Too rapid a growth leads to abuses that damage the image of the sector in the eyes of the political authorities and the public. The sector is thus being singled out, particularly in Belgium. A self-assessment of the risks and a profound work on the sustainability of the industrial activity is urgently needed. On the other hand, trade tensions can appear at world level for exportations to other potato producing zones (recent case in 2020 of import tariffs on the export of European French fries to Latin America or South Africa). So, in the long term, a more geographically diversified potato production within Europe and also worldwide could be suitable to reduce environmental effects such as contribution to greenhouse gas emissions mainly induced through long distance transport, and will contribute to a more regionally shared added value on final potato products.

The seed potato sector in NWEC-05, dominated by the Dutch sector, clearly increased exports over the last decade. NWEC-05 seed potatoes are mainly exported to countries in North Africa, the Middle East and Europe. Destination countries in North Africa and the Middle East often lack the infrastructure to produce and store high-quality seed potatoes, making these countries imports dependent. Within Europe, seed potato exports have benefited from growth in the frozen processed potato industry, which requires specialized potato varieties. A growing global population and increasing demand for frozen processed potatoes will drive the demand for high-quality seed potatoes in the near future. However, opportunities to expand seed potato acreage in the Netherlands are limited. The sector could see a shift to new seed potato-growing areas in NWEC-05 (France, Germany) as already the case with biggest seed companies in the Netherlands, which already produce part of (${~}^{1}\!\left/ \!\!{~}_{3}\right.$) their seed in France, and that could also move to Germany. But high-quality seed production should also be delocalized outside NWEC-05 to other European, Latin America, North African and Asian regions, so that potato production in such regions will be enhanced and contribute more directly to global food security in a more sustainable way. As stated by Devaux et al. (2021), in the mid to long-term enhancement of local and decentralized high quality seed production, multiplication and distribution systems (reducing the dependency on international transports thereby lowering long distance transportation costs whilst reducing the risks associated with the spread of pest and disease), will be suitable option for more sustainability in the global potato production sector. Support to the selection and promotion of locally adapted, demand-led potato varieties, combined with rapid seed multiplication techniques is a corollary.

As already illustrated, a major economic issue of the potato sector is the high volatility in potato price formation versus increasing production costs. For most actors of the production sector in NWEC-05, this will find solution through the setup of national and/or international producers’ organisation (such as the existing NEPG association). This will help to federate their economic influence on the potato markets, together with the setup of national or international interprofessional potato organizations aiming to discuss general potato production and valorization strategy and policy. This will help to mitigate economic issues between potato value chain stakeholders currently based only on contractual policy. This will also ask for a higher level of public support and cooperation inside and between NWEC-05 for common solutions to similar production issues. Such considerations illustrate that there is clearly a huge need for the development of an integrated approach of potato value-chain development with better collaboration between stakeholders’ representatives at NWEC-05 level or even larger (e.g. NEPG, EUPPA, Europatat), but also of national potato organizations such as BELPOTATO.be in Belgium. The main objective of such integrated approaches is still to improve competitiveness and profitability of all the stakeholders of the potato sector whilst mitigating the effects of the current and future challenges such as climate changes, legislation, environmental and economic issues. From an economic point of view, a major objective will be to avoid still increasing production costs because whilst contracted potato prices have increased twice these last two decades this will be unlikely in the coming years and decades.

A last economic issue is that holdings producing potato across NWEC-05 present large diversity in potato cropped areas: large number of holdings with small or intermediary potato areas in France and Germany versus low number in the Netherlands, the UK (with consolidation because of investment required) and Belgium that produces difference in the needs and possibilities for high technologies development (machinery sizing, access and costs for smart agriculture, cost of transfer to high capacities and modern storage facilities). Due to the current expansion trend for potatoes in NWEC-05, the market in the mid to long-term will probably progressively solve this issue, as increase is always going on. But development possibilities and productivity for smaller to medium size and diversified holdings could also be considered, but remain linked to both increasing short potato marketing channels and increasing consumer demand for organic potato production mainly for fresh market.

Finally, consequences of the 2020-2021 COVID-19 pandemic on the potato sector, at least in the short term, will likely affect potato production in NWEC-05 all along the potato value-chain, hampering fresh but also processed potato trade and consumption, and consequently potato demand and production. The main identified factors, linked to COVID-19 pandemic, which negatively affected the NWEC-05 potato sector development mainly in 2020, and to a lesser extent in 2021, were decrease of potato export in volumes and prices, important decrease in global potato consumption mainly in catering sector, rising shipping costs and sharp increase in the costs of vegetable oils, plastic films and cardboard. The global impact of the current health situation on the future of the NWEC-05 potato sector remains difficult to predict.

To conclude, one can summarize that potato production in NWEC-05, as a world potato leader region, is expected to develop in a sustainable way providing a considerable reduction in its environmental footprint, increasing income generation and contributing sustainably to increased food security worldwide. To reach this, as stated by Andrivon (2017) and developed by Devaux et al. (2021), two main options need to be considered: (i) produce more with less through better input management and optimization; (ii) produce just as much but waste less, both before and after harvest through better value chain management, better storage, processing, and marketing operations and responding to increased involvement and awareness of consumers.
